# Molecular Mechanism of Substrate Oxidation in Lytic Polysaccharide Monooxygenases: Insight from Theoretical Investigations

**DOI:** 10.1002/chem.202202379

**Published:** 2022-12-05

**Authors:** Marlisa M. Hagemann, Erik D. Hedegård

**Affiliations:** ^1^ Department of Physics Chemistry and Pharmacy University of Southern Denmark Campusvej 55 5230 Odense Denmark

**Keywords:** biomass degradation, cellulose, copper, lytic polysaccharide monooxygenase, redox enzymes

## Abstract

Lytic polysaccharide monooxygenases (LPMOs) are copper enzymes that today comprise a large enzyme superfamily, grouped into the distinct members AA9–AA17 (with AA12 exempted). The LPMOs have the potential to facilitate the upcycling of biomass waste products by boosting the breakdown of cellulose and other recalcitrant polysaccharides. The cellulose biopolymer is the main component of biomass waste and thus comprises a large, unexploited resource. The LPMOs work through a catalytic, oxidative reaction whose mechanism is still controversial. For instance, the nature of the intermediate performing the oxidative reaction is an open question, and the same holds for the employed co‐substrate. Here we review theoretical investigations addressing these questions. The applied theoretical methods are usually based on quantum mechanics (QM), often combined with molecular mechanics (QM/MM). We discuss advantages and disadvantages of the employed theoretical methods and comment on the interplay between theoretical and experimental results.

## Graphical abstract

**Lytic polysaccharide monooxygenases (LPMO)** are copper dependent enzymes that boost the breakdown of cellulose and other recalcitrant polysaccharides through a catalytic, oxidative reaction. However, the exact mechanism and the true nature of the co‐substrate remain controversial. Herein theoretical investigations of the mechanism with substrate are reviewed and advantages and disadvantages of the employed methods are discussed.

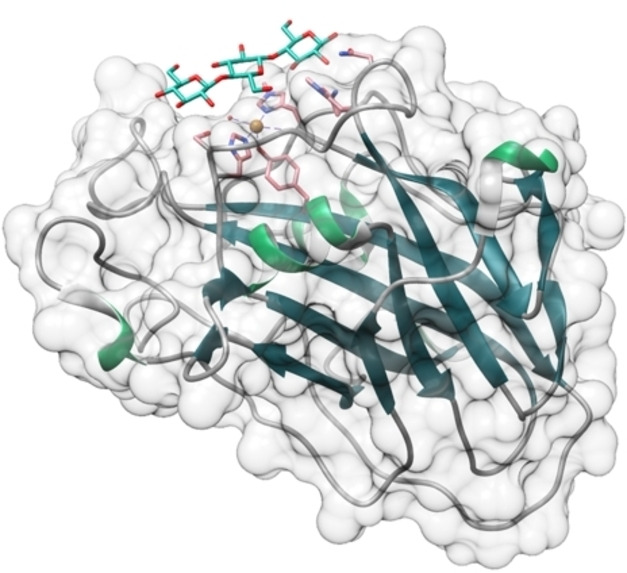

## 1 Introduction

The largest component of biomass waste is the cellulose biopolymer, and this polymer thus comprises an enormous, renewable resource.^[1]^ The breakdown of cellulose (and other polysaccharides) may become an integral part of a more sustainable economy where biomass waste is up‐cycled to higher‐value products. Yet, a remarkable recalcitrance of the cellulose polymer has so far prevented cost‐efficient exploitation.

Cellulose and other recalcitrant polysaccharides are continuously degraded in nature. Until 2010 this was believed to be a slow hydrolytic process.[^2^, ^3^, ^4^] However, this view was challenged with the discovery of metalloenzymes, boosting the process through oxidative chemistry.^[5]^ The responsible enzymes are denoted lytic polysaccharide monooxygenases (LPMOs).[^5^, ^6^] The LPMOs are copper enzymes and comprise a large super‐family, categorized as auxiliary activity^[7]^ (AA) enzymes with the distinct members AA9–AA17 (AA12 is exempted).[^5^, ^6^, ^8^, ^9^, ^10^, ^11^, ^12^, ^13^, ^14^, ^15^] The auxiliary activity refers to a catalytic oxidation of the glycosidic bonds connecting the individual saccharide units in the polysaccharide. This oxidation ultimately leads to disruption of the polysaccharides’ crystalline surface with a concomitant boost in decomposition.[^5^, ^16^] Due to this boost, the LPMOs are now part of commercial enzyme cocktails.[^17^, ^18^] Yet, the mechanism behind LPMOs’ boosting function is still controversial. This is even more intriguing seeing that the scope of the LPMOs continues to expand: the first LPMOs were identified in bacteria^[5]^ and fungi,^[6]^ but LPMOs have since been found in a wide range of host organisms, including oomycetes, algae, viruses, and even complex animals such as arthropods.[^19^, ^20^] Further, LPMOs were recently identified as virulence factors in disease vectors[^21^, ^22^] and agricultural pests.^[15]^ Thus, the enzymes may also have a promising biotechnological potential in agriculture and health sectors.^[19]^ The various LPMOs target a wide range of different polysaccharide substrates with different regio‐ and stereo‐specificities.[^23^, ^24^, ^25^, ^26^]

**Figure 1:**
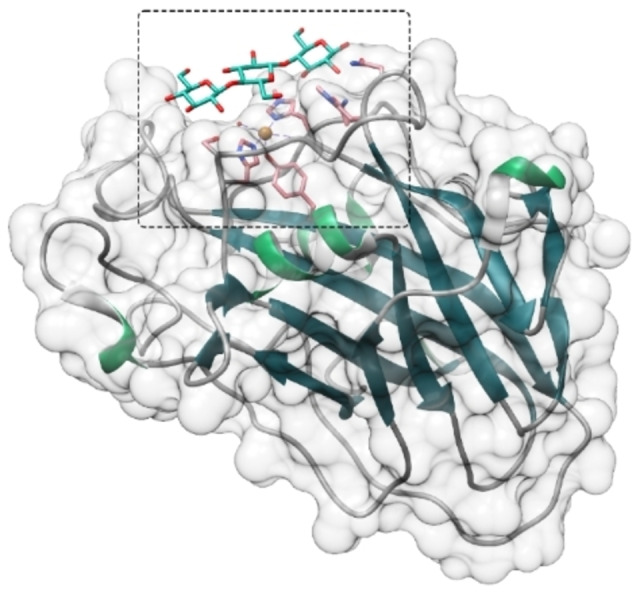
Overall structure of *Ls*(AA9) (PDB: 5ACF^[27]^) as representative LPMO showing the active site (marked by a black square) positioned on a flat surface. Three glucose units are bound on the surface of the enzyme. The active site of this and other, representative LPMOs are presented in more detail in Figure 2.

**Figure 2:**
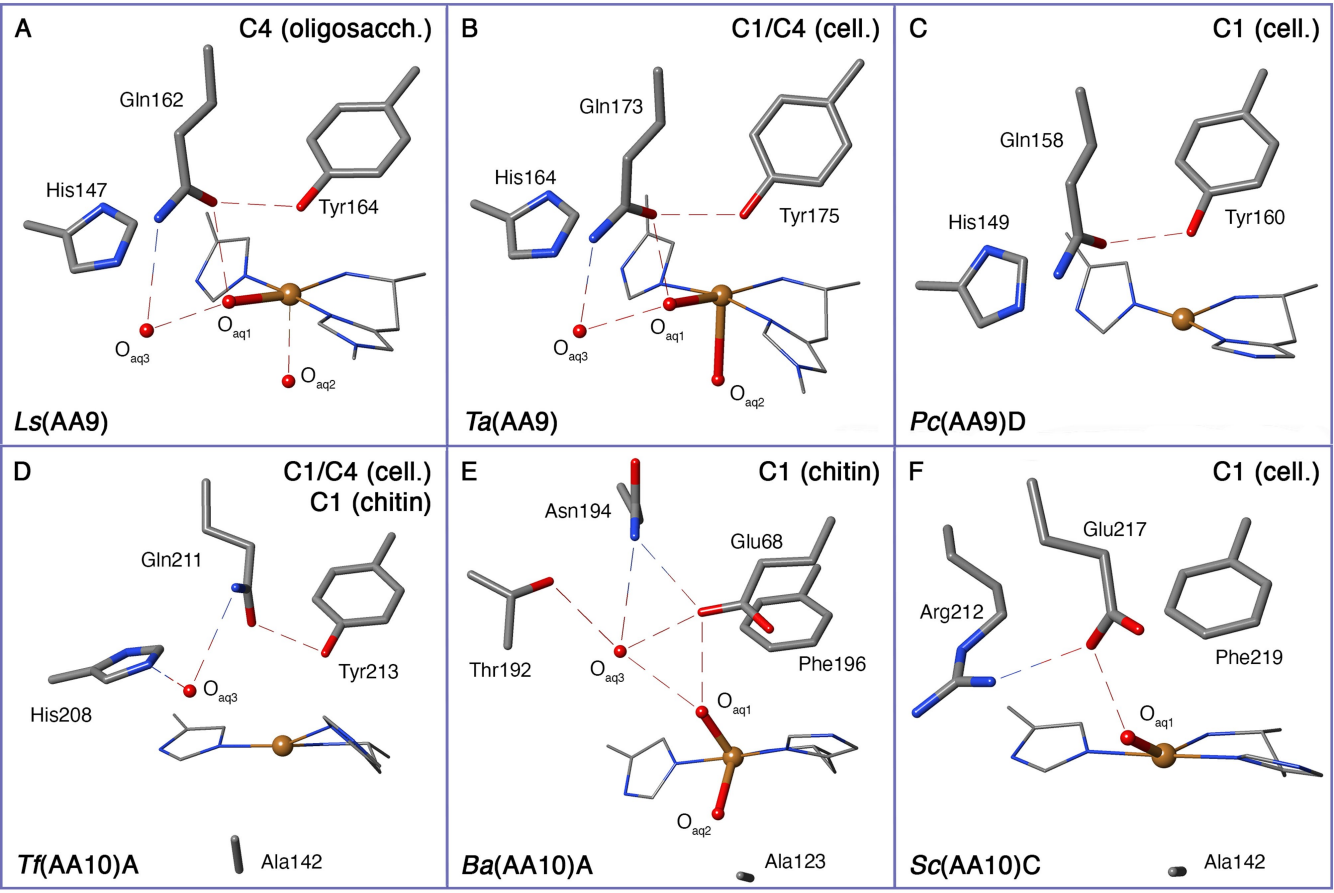
Active sites and important second‐sphere residues of the families AA9 (top) and AA10 (bottom). For simplicity, hydrogen atoms are not shown. The regio‐selectivity of the different LPMOs is provided in the top left corner. The LPMOs are from (PDB IDs are given in parentheses): (A) *Ls*(AA9) (PDB: 5ACG^[27]^). (B) *Ta*(AA9) (2YET^[8]^). (C) *Pc*(AA9)D (PDB:4B5Q^[30]^). (D) *Tf* (AA10)A (PDB: 5UIZ^[31]^). (E) *Ba*(AA10) (5IJU^[32]^). (F) *Sc*(AA10)C (PDB: 4OY7^[33]^). Hydrogen bonds are indicated by dotted lines. O_aq1_–O_aq3_ represent water molecules.

The amino‐acid sequences among LPMOs vary considerably, even within the same family. Still, common features can be discerned: the overall structures of the LPMOs are similar, having similar overall folds and a large, flat substrate‐binding surface.[^28^, ^29^] The copper‐containing active site is located on this binding surface (see Figure 1) and is strictly conserved in all LPMOs. This active site is comprised of two coordinating histidine residues, in which one histidine (the amino‐terminal residue) coordinates bidentate through the N‐terminus and the imidazole side chain. This has become known as the histidine brace.^[8]^ Active sites for selected LPMOs from the AA9 and AA10 families are shown in Figure 2.

This figure also includes part of the second‐coordination sphere that can differ significantly for different LPMOs. The differences include even residues coordinated to the Cu ion: the tyrosine residue seen in Figure 2A–D is in most AA10 LPMOs replaced with phenylalanine, as seen in Figures 2E and F. However, AA10 LPMOs with a coordinating tyrosine are also known. The AA10 LPMOs also have a conserved alanine in the second‐coordination sphere (Figures 2E–F), although a recently characterized AA10 LPMO, *Pl*(AA10), has the alanine replaced by isoleucine.^[34]^

The steric constraints of the alanine/isoleucine residue typically lead to a five coordinated (trigonal bipyramidal) structure of the first coordination sphere in AA10s, compared to the pseudo‐octahedral sphere of Cu(II) in AA9s.[^33^, ^35^, ^36^, ^37^, ^38^] Another difference between AA9 and AA10 LPMOs is that the terminal histidine in Figures 2A and B is N‐methylated, which is the case in most AA9 LPMOs^[39]^ (Figure 2C shows an exception). The AA10 LPMOs generally do not have this methylation (cf. Figures 2D–F) and its’ role has remained somewhat mysterious. A possibility is that it increases the enzymes’ resistance to oxidative damage.^[40]^

The active sites displayed in Figure 2 are all from crystal structures, where photo‐reduction of the Cu(II) ions in the X‐ray beam^[41]^ leads to structures displaying a mixture of Cu(I) and Cu(II) oxidation states. We here assume that the active sites with two coordinating water molecules predominantly contain Cu(II). In LPMOs with Cu(II) and a coordinating tyrosine, the Cu−O bonds to tyrosine and the water molecule *trans* to the tyrosine (O_aq2_ in Figure 2) are usually elongated due to an axial Jahn‐Teller distortion.^[42]^ Accordingly, O_aq2_ is denoted the *axial* water, whereas O_aq1_ is denoted the *equatorial* water. Finally, we note that the second‐sphere histidine (His) and glutamine (Gln) shown in Figures 2A–D are speculated from mutation studies^[43]^ to be part of the mechanism, but they are not conserved over all LPMOs as seen from Figures 2D and E. We comment on possible roles for these residues later, when comparing experimental studies to the calculated mechanisms.

**Figure 3:**
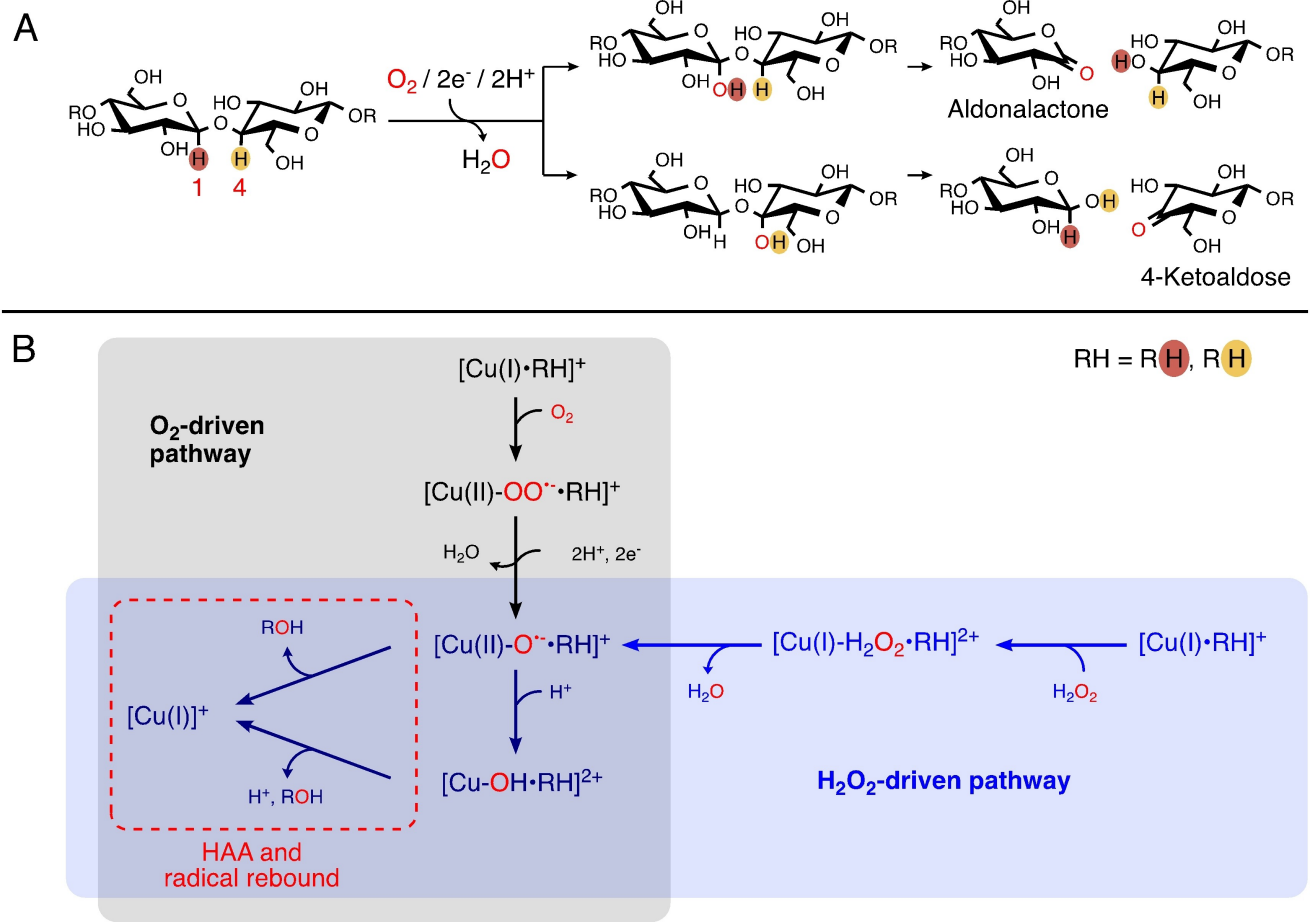
A) Oxidative cellulose degradation catalyzed by LPMOs. The C_1_ and C_4_ hydrogen atoms are highlighted in red and yellow, respectively. C_1_ and C_4_ positions are marked red. B) Simplified mechanism of the two possible pathways for hydrogen atom abstraction (HAA) from C_1_ or C_4_ depending on the co‐substrate. The scheme excludes the initial priming reduction of Cu(II)‐LPMO to obtain the catalytically active Cu(I)‐LPMO.

The overall oxidation reaction performed by LPMOs is shown in Figure 3A, using cellulose as an example: the LPMOs oxidize the polysaccharides at one of the two carbon atoms involved in the glycosidic bond (C_1_ or C_4_). Whether C_1_ or C_4_ is oxidized is highly dependent on the specific LPMO and some LPMOs can also target both carbon atoms. This regio‐selectivity has for AA9 LPMOs led to a sub‐classification into type 1 (oxidize C_1_), type 2 (oxidize C_4_), and type 3 that can oxidize both atoms.[^10^, ^33^, ^44^, ^45^] One of each type is shown in Figure 2A–C.

The nature of the co‐substrate has been discussed intensely in recent years: originally, the co‐substrate was believed to be O_2_,^[5]^ but more recent experimental investigations have suggested both O_2_ and H_2_O_2_ or only H_2_O_2_ as the natural co‐substrate(s).[^46^, ^47^] A simplified mechanism showing the putative pathway with both co‐substrates is included in Figure 3B. In a later figure, we provide a more detailed mechanistic picture. Several reviews concerning different aspects of this mechanism have been published.[^2^, ^17^, ^18^, ^29^, ^38^, ^39^, ^42^, ^44^, ^48^, ^49^, ^50^, ^51^, ^52^, ^53^, ^54^, ^55^, ^56^, ^57^, ^58^, ^59^, ^60^, ^61^] Here we exclusively focus on the mechanism for the oxidative reactivity with the polysaccharide substrate. This part is still not clarified, despite significant effort from both theoretical and experimental groups alike.[^28^, ^38^, ^44^, ^49^, ^53^, ^62^, ^63^, ^64^, ^65^, ^66^, ^67^] Although we here focus primarily on results from theoretical models, we will often compare the theoretical results to experimental investigations. Our focus on substrate oxidation means that we only treat the substrate binding process superficially (we refer instead to the previous reviews[^29^, ^39^, ^42^, ^49^] for a more thorough discussion of this part).

The employed theoretical methods also possess inherent challenges, and we critically comment on these challenges throughout the review. After an introduction to the most commonly employed theoretical methods, we give a brief overview of the mechanism, before discussing the individual steps and intermediates in more detail.

## 2 Theoretical Methods

The substrate oxidation mechanism inevitably involves forming and breaking of chemical bonds. For this situation, computational modeling of chemical systems generally requires a method founded in quantum mechanics (QM). Except one study,^[68]^ all QM investigations of LPMOs employ density functional theory (DFT). We describe both merits and disadvantages of DFT below.

**Density functional theory**: in the Kohn‐Sham formulation is currently the most widely applied QM approach for modeling bio‐inorganic chemistry.[^69^, ^70^] The methodology often provides sufficiently accurate results for comparatively large systems (*>*1000 atoms) at a relatively low computational cost. This favorable performance is achieved through the treatment of electron correlation with a so‐called correlation‐exchange functional. This treatment is computationally much faster than many other QM methods. Unfortunately, the method does not recover electron correlation systematically, leading to a highly system dependent performance. For instance, obtaining relative energies of different spin‐states have been an issue, which has affected calculations on LPMOs: the choice of functional can significantly alter spin‐state and reaction energetics for certain reaction intermediates in the LPMO catalytic cycle.^[66]^ Calculations with highly accurate wave functions indicate that these intermediates are problematic for current DFT methods.^[68]^

Despite the efficient treatment of electron correlation, the computational scaling of DFT still does not allow calculations on full LPMO enzymes. The first investigations therefore employed only a small cluster around the active site.^[62]^ The remaining protein is either ignored or described with a so‐called continuum model,^[78]^ originally developed to mimic a solvent using the dielectric constant (*ϵ*) as input. It has been a general procedure for metalloenzymes to employ a small cluster, combined with a very low dielectric constant to mimic the protein (although this approach may be questionable for an active site very close to the surface as seen in LPMOs). We denote this method a QM‐cluster method. Note that we use this term regardless of whether a continuum model is employed or not. We discuss methods to address the poor scaling of QM methods below.

**Molecular mechanics** builds on classical mechanics and describes atoms as bonded together with (classical) harmonic forces (springs). Thus, molecular mechanics (MM) methods can avoid the poor scaling of QM methods. However, they employ heavily parameterized expressions to compensate for the lacking QM description (and the parameterization of transition metals is exceedingly difficult). Further, describing bonds by a harmonic force, makes MM methods unsuitable for studying bond breaking. Nevertheless, with proper parameterization they can be made reasonably accurate and very efficient for equilibrium structures. They are therefore often used to investigate the dynamics of biomolecular systems in molecular dynamics (MD) simulations^[70]^ (see at the end of this subsection).

**QM/MM hybrid methods** exploits that enzymes usually perform chemical reactions in a rather small region (i.e., at the active site) of the full enzyme.[^79^, ^80^] Thus, we can employ a QM method (usually DFT) for the active site, while a less accurate, but efficient, MM treatment is used for the remaining system.^[79]^ Compared to the QM‐cluster approach, the enzyme is thus still treated explicitly in QM/MM, and not ignored or replaced by a structure‐less continuum.

Selection of a proper QM region is a specific challenge for QM/MM: calculated QM/MM energies tend to converge rather slowly with the QM system size. In other metalloenzymes, systematic investigations of the QM size have shown that QM/MM reaction energies can be very sensitive to the QM region size.[^81^, ^82^] This may also be the case for LPMOs, where second‐sphere residues are likely to be important for the mechanism.^[43]^ A set of rules to define a sufficiently large QM region was defined for [NiFe]‐hydrogenases[^81^, ^82^] (denoted the Big‐QM method), but the resulting QM regions become very large.[^82^, ^83^] The Big‐QM method has been employed for LPMOs^[72]^ but not for investigations of the substrate oxidation mechanism.

**Molecular dynamics**: Static QM/MM methods relying on one (or a few) structure(s) may provide valuable information for the characterization of a reaction. However, biomolecular systems such as LPMOs are naturally dynamic systems, and many thermally accessible conformations may contribute to the mechanism. Such conformations can be sampled by including the system dynamics and free energy barriers of enzymatic reactions can depend significantly on this sampling.^[84]^ The dynamics can be included by handling the nuclei as classical particles (Newtons’ second equation) and iteratively obtain the time‐dependent motion (trajectories). This will naturally require an underlying energy function, where either an MM force field or a QM/MM method can be employed. We denote the former method as a classical MD and the latter as a QM/MM MD (although both methods treat the dynamics classically).

For LPMOs classical MD has been used to investigate the binding of substrate to the LPMO involving free‐energy methods.[^30^, ^74^, ^75^] The time scale of this reaction, combined with the stability of the involved intermediates, has allowed extensive interplay between the classical MDs and various experimental techniques. This is briefly surveyed in the next section. The QM/MM investigations in Table 1 also employ MD to equilibrate the system (e.g., through simulated annealing^[84]^), but these MDs are generally short. In one case, also a longer MD was employed,^[65]^ but only one structure was extracted for QM/MM (cf. Table 1).

**Table 1:** Theoretical studies on the LPMOs mechanism. Truncated substrate is Gly_2_ or NAG_2_ and H_2_O_ax._, H_2_O_eq._ refer to the active site water molecules. Note that a few of the theoretical investigations in the table investigate different parts of the mechanism than substrate oxidation (they are included as they are used in the discussion). A recent QM‐cluster and MD investigation^[75]^ is not included as it was unclear whether they report free energies or electronic energies.

LPMO	Intermediates	Methods	Setup and comments	Functional	QM region
1. *Ta*(AA9)A	**1**, **2**, **3 a**, **6 b** ^[62]^	QM‐cluster (CPCM ϵ =4.3)	Starting from a crystal structure (PDB 2YET) the substrate was added, and selected distances were kept fixed during optimization (different starting geometries for the enzyme‐substrate complex were taken from MD simulations of a similar LPMO^[30]^).	B3LYP, M06‐L and B3LYP‐D3	Cu, His1, His86, His164, Gln173, Tyr175, 3xH_2_O, O_2_/OOH/O, Gly_2_
2. *Ta*(AA9)A	**3 a** ^[71]^	QM‐cluster (PCM ϵ =4.0)	Starting from a crystal structure (PDB 3ZUD), truncated systems were created with selected atoms frozen during optimizations.	B3LYP	Cu, His1, Ser85, His86, His164, Gln173, Tyr175, H_2_O_ax._, H_2_O_eq._/O_2_
3. *Ta*(AA9)A	**1**, **2**, **3 a** ^[72]^	QM/MM (DFT), Big‐QM (DFT and COSMO ϵ =4.0)	Equilibration was done starting from a crystal structure (PDB 2YET). The MM part was either kept fixed or optimized on the MM level within 6 Å of the QM region.	TPSS‐D3, B3LYP‐D3	Cu, His1, His86, Tyr175, H_2_O_ax._, H_2_O_eq._/O_2_
4. *Ls*(AA9)A	**3 a**, (**3 b**), **4 a**, (**4 b**), **6 a**, **6 b** ^[63]^	QM/MM (DFT), QM cluster (DFT and COSMO, ϵ =4.0)	The starting structures were obtained from QM/MM optimisation of **3 a** (see entry 6). The BDE calculations were from QM‐cluster calculations with a truncated cluster, freezing selected atoms (Thr2 and the substrate were removed for the cluster calculations).	TPSS‐D3, B3LYP‐D3	Cu, His1, His78, Tyr164, Thr2, O_2_/OOH/O, Gly_2_
5. *Nc*(AA9)	**1**, **2**, **3 a**, **4 a**, **4 b**, **5 a**, **6 a**, **6 b** ^[64]^	QM‐cluster	A truncated crystal structure (4EIS, chain B) was used as starting structure with an added celloheptose unit and chosen atoms were kept fixed (no constraints on the polysaccharide atoms). The model was validated by comparing the optimized Cu(II) and Cu(I) geometries with the experimental data. A smaller model is employed for TS optimization (His1, His82, and Tyr171), and the cellobiose as substrate); the resulting structure is re‐inserted into the large model and optimized while freezing atoms that moved along the reaction coordinates.	BP86, B3LYP	Large model including Cu, first‐coordination‐sphere residues (His1, His82, Tyr171, H_2_O_ax._), 14 additional ‘second shell’ residues, cellulose oligomer, two side‐chain tyrosinases
6. *Ls*(AA9)A	**3 a**, **4 a**, **4 b**, **6 a**, **6 b**, **7 a** ^[66]^	QM/MM (DFT)	A crystal structure of a LPMO‐substrate complex (PDB 5ACF) was solvated and equilibrated before QM/MM optimisation. The MM part was either kept fixed or was optimized on MM level (within 6 Å of the QM region). The TS were found by linear transit calculations.	TPSS‐D3, B3LYP‐D3	His1, His78, His147, Thr2, Cu, (2×H_2_O added for the H_2_O_2_ pathway).
7. *Ls*(AA9)A	**5 a**, **5 b**, **6 c** ^[65]^	QM‐cluster (DFT), QM/MM (DFT), HCC (SMD solvation model)	A crystal structure of a LPMO‐substrate complex (PDB 5ACF) was used to construct QM‐cluster models (here frequency calculations are used to verify TS and minima structures as well as for thermochemical corrections). For QM/MM, equilibration, and MDs (50 ns) were done prior to QM/MM optimization (using a single, representative, snapshot). TSs were located by PES scans followed by full TS optimizations. Note that they report electronic energies for the QM/MM calculations. Gibbs free energies for the hydrolysis of the C4‐hydroxylated intermediate in water solution were estimated using cluster calculations with the BMK functional.	B3LYP, BMK for hydrolysis in HCC calculations.	QM/MM: His1, His78, His147, Glu148, Gln162, Cu, H_2_O_2_, 3 units of oligosaccharide. Small cluster: Cu, His1, His122, Tyr164, Gln162, 1 glucose unit, solvent water
8. *Jd*(AA10)A	**3 a** ^[73]^	Quantum refinement, QM‐cluster (DFT and COSMO, ϵ =80), QM/MM	A QM‐refined X‐ray strcuture (from PDB 5VGO) was used as starting structure for equilibration, followed by QM/MM optimization (with fixed MM).	TPSS‐D3	His109, His32, Phe164, WAT307, Cu, O_2_, Glu65, additional solvent molecules
9. *Sm*(AA10)A	**5 a**, **5 b**, **6 b** ^[67]^	QM/MM/MD (DFT), QM/MM (DFT)	The system was set up from a previous computational (QM/MM MD) study of a substrate complex, starting from a crystal structure (PDB 2BEM:C^[56]^). Here, QM/MM optimizations were done after 200 ns equilibration^[74]^ of **1** (with chitin). Further MDs (50 ns) were carried out from this with a H_2_O replaced by H_2_O_2_ (the H_2_O_2_ was restrained close to Cu) and a small QM region. One snapshot was then QM/MM optimized (with a larger QM region), yielding **5 a**. The mechanism was studied with outset in **5 a**.	TPSSh‐D3, BP86‐D3, B3LYP‐D3	Cu(I), His28, His114, Glu60, NAG${}_{2}$
10. *Ls*(AA9)A	**1**, **2**, **3 a**, **4 b**, **6 b** ^[76]^	QM/MM MD and meta‐dynamics	The setup was similar to entry 7, but with the substrate removed from the structure (PDB 5ACF). Tailored MM force fields were employed for intermediates with [CuO_2_]^+^ and CuOOH]^+^ moieties (correspondig to **3 a** and **4 b** without substrate).	B3LYP	Cu, His1, His78, Tyr164, Gln162, AscH/Substrate, active site water molecule, oxygen moiety
11. *Ls*(AA9)A	**6 b**, **7 a** ${}^{'}$ ^[77]^	QM/MM, TD‐DFT	Setup from a previous computation (QM/MM) study of a substrate complex was used (see entry 6).	TPSS‐D3, B3LYP‐D3, CAM‐B3LYP	Cu, Hi, Thr2, His147, Gln162, Tyr164, H_2_O_ax._, H_2_O_eq._/O/OH (the TD‐DFT system was further truncated, excluding Thr2)

When it comes to QM/MM MD, the simulations for systems of the size of LPMOs are computationally expensive. This leads to time‐scale problems, both if the goal is to sample conformations, but also for reactions (which is a relatively rare event that requires extensive sampling of the potential energy surface). For reactions, enhanced sampling techniques (e.g. umbrella sampling^[85]^ and metadynamics^[86]^) are therefore required. A few QM/MM MD investigations of LPMOs have appeared recently,[^67^, ^76^, ^87^] but only Ref. [67] investigated substrate oxidation. The two other investigations focus on the formation of H_2_O_2_ ^[76]^ and electron transfer during the reduction of the resting state,^[87]^ respectively.

Note that the QM/MM MD investigations only employ a single structure (snapshot) from the QM/MM MD and generally employ time scales too short for proper sampling of the enzyme. Proper sampling is generally a large challenge for QM/MM MDs of enzymatic reactions (see Ref. [88] for a recent discussion).

## 3 An Overview of the Substrate Oxidation Mechanism

**Figure 4:**
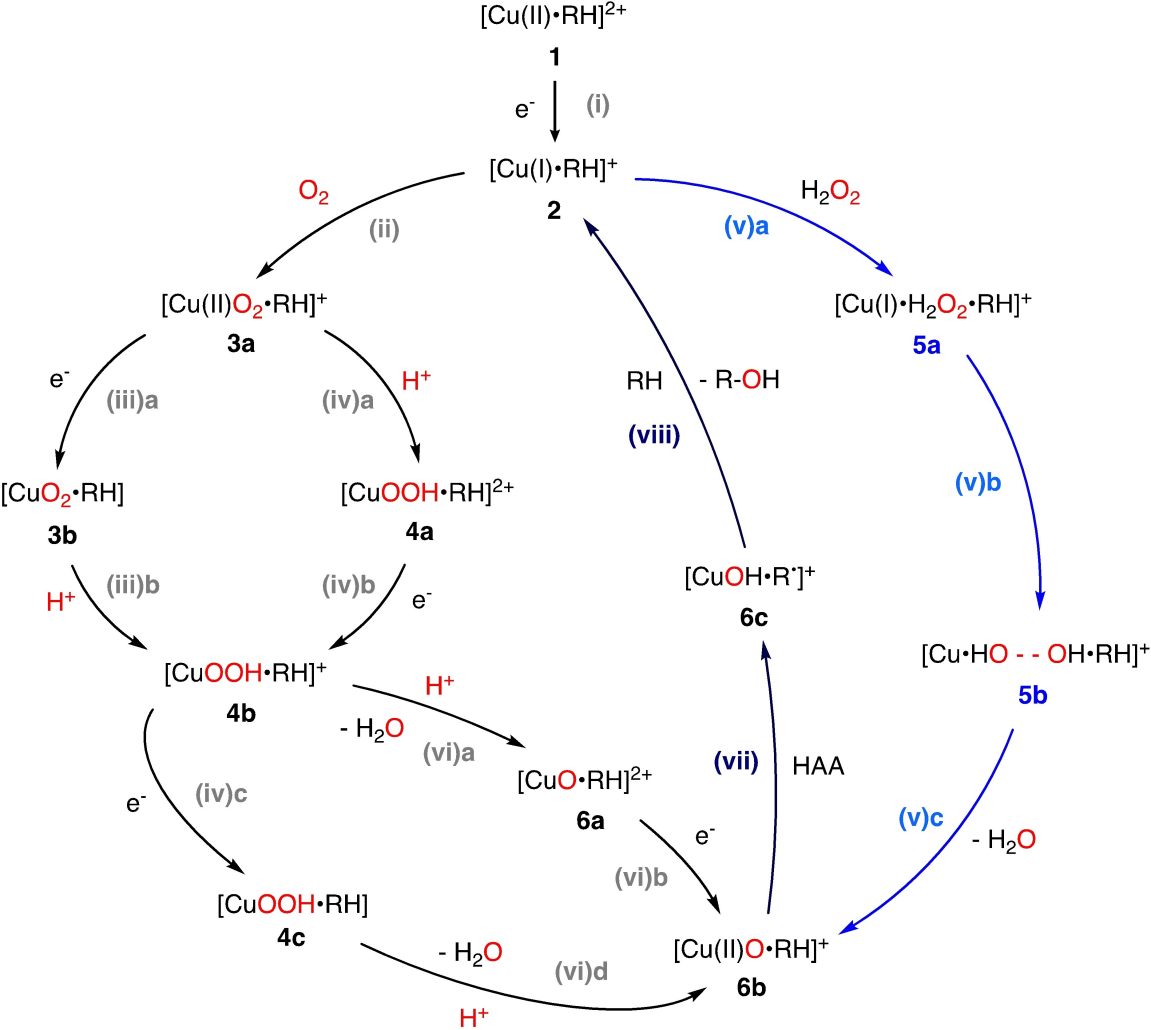
Schematic summary of the putative mechanisms for the HAA by LPMOs for either the O_2_‐driven (left) or the H_2_O_2_‐driven reaction (blue, right). The substrate is denoted RH. The figure is primarily based on the investigations in Refs. [62, 64–67].

**Figure 5:**
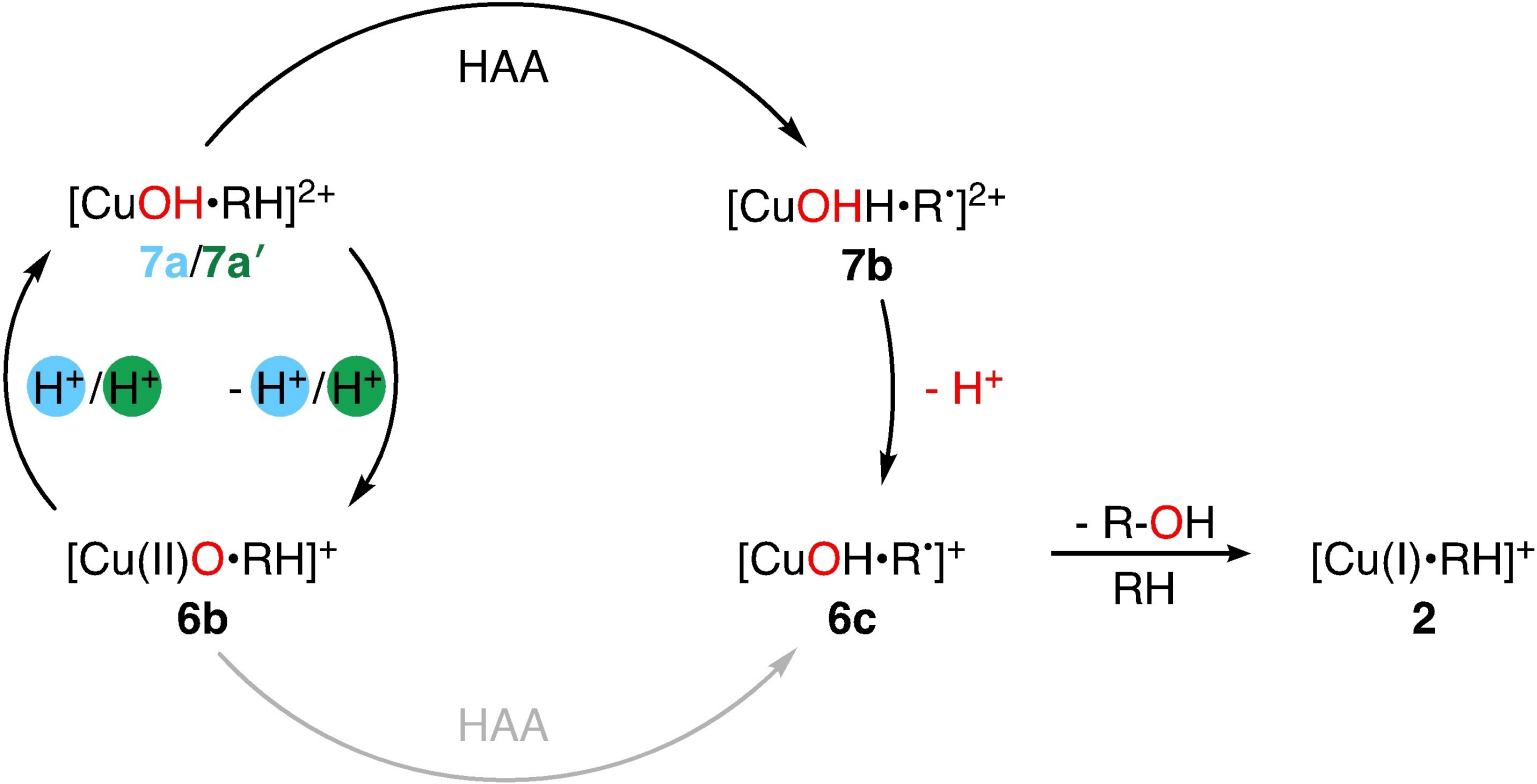
Alternative pathway for HAA if the [CuO]^+^ (**6 b**) intermediate from Figure 4 is protonated. Different proton sources lead to **7 a** (His) and **7 a** ${}^{'}$ (Tyr), respectively. The substrate is denoted RH. The figure is primarily based on the investigations in Refs. [66, 77].

We now discuss the LPMO substrate oxidation mechanism in more detail. While Figure 3B provides a simplified overview of the mechanism, a detailed overview of the different mechanistic proposals is given in Figure 4 and Figure 5. We have in these figures labeled the various intermediates **1**–**7**, and we use these labels in the following. The oxidation states of copper are somewhat speculative after O_2_ or H_2_O_2_ has been introduced, and they are only given in Figures 4 and 5 for a few intermediates where they have been investigated thoroughly.

Progress in elucidating the mechanism has been somewhat hampered by the fact that most LPMOs target insoluble polysaccharide substrates, which complicates many experimental methods. Thus, Figures 4 and 5 are constructed based on previous theoretical investigations of the full mechanism.[^62^, ^64^, ^65^, ^66^, ^67^] However, the different investigations employ different methods, systems sizes, and different underlying LPMOs. This complicates the comparison between the theoretical investigations, and we have therefore summarized the key computational parameters for the individual studies in Table 1. This table also indicates which intermediates that are included in each study. We have divided Figure 4 into a number of reaction steps, *(i)*–*(viii)*. We discuss the individual steps below. A few alternative suggestions for intermediates with potential involvement in HAA from the substrate are given in Figure 5 and they are discussed last.

**Step (i). The initial reduction**: In the first step, the Cu(II) ion in the active site of intermediate **1** is reduced to Cu(I). It is now established that catalytic turnover requires a ‘priming electron’ to reduce Cu(II) to Cu(I).^[17]^ A number of different electron donors have been employed, either small molecules or the enzyme cellobiose dehydrogenase (CDH).[^5^, ^11^, ^65^, ^76^, ^89^, ^90^, ^91^, ^92^, ^93^] Note that we in Figure 4 have assumed the substrate (RH) to be bound already in **1**, although it is not clarified whether reduction occurs before or after substrate binding.^[94]^ There is some evidence that substrates tend to bind better after reduction[^94^, ^95^, ^96^] of the resting state (the resting state corresponds to **1** without the substrate).

The substrate binding process has been subject to extensive investigations and some of the residues involved may also determine whether the LPMO oxidize C_1_ or C_4_.^[29]^ As earlier noted, this review focuses on the potential oxidative intermediates performing the HAA from the substrate, rather than the substrate binding. However, we still give a brief overview of experimental and theoretical results for substrate binding here.

Crystallographic studies of substrate‐bound LPMOs are rare and only exist for two closely related AA9 LPMOs, *Cv*(AA9) and *Ls*(AA9)[^27^, ^97^] of which the latter is shown Figure 1. A crystallographic investigation was only possible since these LPMOs can bind smaller, soluble oligosaccharides.^[29]^ The X‐ray structure shows that the axial water molecule (cf. Figure 2) in the resting state (i.e., **1** without substrate) is usually displaced upon substrate binding. The substrate‐bound LPMOs always have a Cl^−^ ion from the crystallization buffer replacing the equatorial water ligand. The binding of Cl^−^, as well as the always present photo‐reduction,^[29]^ complicate direct comparison to theoretical studies, where the Cl^–^ ion is removed.

The X‐ray photo‐reduction was investigated very recently by Tandrup et al.^[98]^ in a systematic study of two AA9 LPMOs, namely *Ls*(AA9) and *Ta*(AA9). Tandrup et al.^[98]^ obtained structures where Cu(II) was increasingly photo‐reduced by gradually increasing the X‐ray dose. This led to structures with increasingly dissociated water ligands. This confirmed a previous investigation by Gudmundsson et al.^[99]^ for an AA10 LPMO. Reducing the substrate‐bound *Ls*(AA9) LPMO leads to dissociation of the Cl^−^ ligand at high X‐ray dose.^[98]^ This can be compared to the QM‐cluster and QM/MM structure optimizations, which show that the reduction is associated with dissociation of the equatorial water molecule.[^66^, ^71^] A recent paper by Theibich et al.^[100]^ compared QM/MM and X‐ray structures, which showed that QM/MM qualitatively reproduces changes induced by substrate binding (unfortunately, no RMSD values were reported). Comparing the RMSD value between optimized and X‐ray structures has been employed as a quality measure for QM‐cluster calculations,[^30^, ^71^] but more systematic investigations could provide important quality measures in future studies. With the results in Ref. [98], we now have a stepping‐stone for such benchmark calculations.

Investigations of substrate binding have, unlike the steps in the mechanism after O_2_/H_2_O_2_ binding, employed more extensive MD methods (mostly with classical MM). Typically, this has involved calculations of the free‐energy change for the substrate binding process.[^30^, ^74^, ^75^] This has also been combined with well‐established experimental methods such as site‐directed mutagenesis,[^6^, ^101^, ^102^] where amino acids that are important for substrate binding can be singled out. A recent investigation employed this strategy for an AA9 LPMO and found correlation between the change in interaction energy for specific residues and mutated residues. Thereby, residues that are crucial for the LPMO‐substrate interaction can be identified.^[75]^ The binding process has also been studied directly by nuclear magnetic resonance (NMR)[^103^, ^104^] (typically Zn‐loaded or apoproteins are used since copper can obscure the NMR spectra). Bissaro et al.^[74]^ employed extensive MD simulations on chitin binding to an AA10 LPMO: the residues with high energy contributions correlated with NMR^[103]^ as well as site‐directed mutagenesis experiments.^[101]^

As a complement to NMR, a few investigations have also employed electronic paramagnetic resonance (EPR) spectroscopy, which can probe specific active site changes around the Cu(II) ion upon substrate binding.[^27^, ^105^, ^106^] For instance, the axial water displacement upon substrate binding seen in crystallographic work^[27]^ was confirmed (along with other modifications of the active site) since it was reflected in the obtained spin‐Hamiltonian parameters.[^27^, ^42^, ^105^] In recent papers, calculated spin‐Hamiltonian parameters were compared with the experimental ones.[^74^, ^100^, ^106^, ^107^] A qualitative agreement was achieved, but the EPR parameters are infamous for being very dependent on the computational setup. We refer to Refs.^[100]^ for a detailed discussion.

The above investigations, combining site‐directed and spectroscopic results with MD simulations, provide a setup to critically evaluate the calculated energetics. In turn, this can also validate experimental conclusions. Thus, the substrate binding part of the mechanism illustrates excellently the interplay of theoretical and experimental methods to gain molecular insight. Unfortunately, many of the methods cannot be employed as easily after introduction of O_2_/H_2_O_2_ since the formed intermediates are much more reactive. Therefore, the later steps in the mechanism discussed below are more difficult to validate. Some kinetic data exist, but they are known to be highly dependent on the reaction conditions. We return to this discussion in the sections on the reaction intermediates.

**Steps (ii)–(vi). Co‐substrate binding**: After reduction (and possibly substrate binding), the LPMO can interact with a co‐substrate. In Figure 4, we have included pathways with both O_2_ and H_2_O_2_, although most of the theoretical investigations that formed the basis for the figure include only one of the pathways.

The steps *(ii)*–*(iv)* are for the O_2_ pathway, where step *(ii)* is the binding of O_2_, leading to an intermediate with a [CuO_2_]^+^ moiety (**3 a**). Although reduction seems to be a pre‐requisite for O_2_ binding, a recent QM/MM MD study^[87]^ suggests that O_2_ binding can be coupled with an interprotein, long‐range ET from CDH to Cu(II)‐LPMO. Thereby a [CuO_2_]^+^ species is formed, bypassing the formation of a Cu(I)‐LPMO. The formed species corresponds to **3 a** in Figure 4, but without substrate. The introduction of substrate will potentially block the efficient ET pathway, but this has not yet been investigated.

Reduction of **3 a** in step *(iii)a* gives the intermediate [CuO_2_] (**3 b**), whereas protonation in step *(iv)a* yields a [CuOOH]^2+^ (**4 a**) intermediate. An intermediate with [CuOOH]^+^ moiety (**4 b**) can thus be obtained from either protonation of **3 a** as in step *(iv)b* or reduction of **4 a** as in step *(iii)b*. Further reduction of **4 b** in step *(iv)c* leads to an intermediate with a [CuOOH] moiety (**4 c**). We can from **4 b** under addition of a proton and an electron (either concerted or in steps) obtain the [CuO]^+^ (**6 b**) intermediate in which the oxygen O−O bond is cleaved. Thus, steps *(vi)a* and *(vi)b* and *(iv)c* and *(vi)b* from **4 b** both lead to **6 b**, but differ in the order in which the electrons and protons are added.

The H_2_O_2_ pathway is marked in blue in Figure 4. In this pathway, H_2_O_2_ is added to intermediate **2** in step *(v)a*, binding loosely to the active site through second‐sphere residues. We denote the resulting intermediate **5 a** in Figure 4. Several investigations[^65^, ^66^, ^67^] have shown that reaction *(v)b* where H_2_O_2_ reacts with Cu(I) in **5 a**, leads to an intermediate with an elongated O−O bond (**5 b**).[^65^, ^66^, ^67^] From this, the oxyl [CuO]^+^ (**6 b**) intermediate can be formed directly under formation of H_2_O as shown in reaction *(v)c*. Importantly, this means that the formation of an [CuO]^+^ (**6 b**) intermediate, unlike in the O_2_‐driven pathway, can occur without exogenous electrons and protons, which in the O_2_‐driven pathway would have to be transferred to the active site, while the substrate is bound.

**Step (vii). Hydrogen atom abstraction from the substrate**: The [CuO]^+^ (**6 b**)[^49^, ^53^, ^64^, ^72^, ^108^] and [CuO_2_]^+^ (**3 a**),[^45^, ^49^, ^91^, ^109^] intermediates are the intermediates most frequently investigated as the species performing HAA from the substrate (this step is marked with HAA in Figure 4 – note that only HAA from **6 b** is shown). The resulting intermediate where a hydrogen has been abstracted is denoted as [CuOH ⋅ R⋅]^+^ (**6 c**) in Figure 4. The intermediates **3 a** and **6 b** have been thoroughly analyzed and both their spin‐states and electronic structure indicate a superoxide or oxyl bound to a Cu(II) ion.[^62^, ^64^, ^65^, ^66^, ^71^, ^72^]

The first theoretical study on the oxidative mechanism of LPMOs by Kim et al.^[62]^ focused exclusively on the O_2_‐driven pathway (the study was published before the discovery of the H_2_O_2_‐driven pathway). They employ a QM‐cluster approach similar to a later investigation by Bertini et al.^[64]^ However, the employed functionals, cluster sizes, and underlying LPMOs are different in the two studies (albeit both are AA9 LPMOs). Further, Bertini et al.^[64]^ also investigated the H_2_O_2_ pathway. Two subsequent QM/MM investigations by Hedegård and Ryde^[66]^ and Wang et al.^[65]^ also investigated the H_2_O_2_ pathway; the former study compared both O_2_ and H_2_O_2_ pathways. We will later compare these two investigations in more detail, as they employed the same underlying *Ls*(AA9) LPMO (cf. Table 1). The paper by Wang et al.^[65]^ in addition to QM/MM also reports results from QM‐cluster calculations with small QM regions, and we occasionally discuss these results as well.

**Step (viii). Recombination**: The step after the HAA in Figure 4 is a so‐called recombination step: the radical substrate (R⋅ in intermediate **6 c**) is recombined with OH, bound to Cu. We here focus less on this reaction set step since it, compared to the HAA step, has smaller activation barriers and very favorable reaction energies.[^62^, ^64^, ^66^, ^67^] The recombination step is therefore combined with the release of the substrate to regenerate **2** in Figure 4.

**Alternative intermediates for hydrogen atom abstraction**: In addition to Figure 4, we have included an additional set of potential oxidative intermediates in a separate figure (Figure 5). They can be seen as a protonated form of the [CuO]^+^ (**6 b**) intermediate. We present these protonated forms separately to emphasize that different protonation sources have been employed: either a second‐sphere histidine^[66]^ or the axial tyrosine.^[77]^ We denote the resulting [CuOH]^2+^ moiety **7 a** in the former case, and **7 a** ${}^{'}$ in the latter case; both options are collected in Figure 5. The intermediates with a [CuOH]^2+^ moiety have not been under same scrutiny as the [CuO]^+^ (**6 b**) and [CuO_2_]^+^ (**3 a**) intermediates. We therefore do not show oxidation states for the Cu ion in Figure 5. The oxidation states are also ambiguous, seeing that the [CuOH]^2+^ moiety can be interpreted as either a Cu(II)‐hydroxyl or a Cu(III)‐hydroxide species.[^66^, ^72^] Moreover, in the case of tyrosine deprotonation, it is likely that a tyrosine radical is obtained[^77^, ^110^, ^111^] further obscuring the oxidation state.

In inorganic model systems resembling the LPMOs active site it has been shown that Cu(III)− hydroxy species can carry out HAA from C−H bonds.[^112^, ^113^, ^114^, ^115^] In many of these investigations, the oxidative power is estimated from bond dissociation energies (BDEs).[^114^, ^115^, ^116^, ^117^, ^118^, ^119^, ^120^, ^121^] The background for using the BDE as a measure for an intermediates’ oxidative power is that the HAA reaction energy can be understood as the energy difference given in equation 1

(1) $$\;\Delta E_{react.}=\Delta E_{R-H}^{BDE}-E_{{\left[Cu-XH\right]}^{n+}.}^{BDE}\;$$

The BDE energies above denote the energy required to abstract hydrogen from the substrate (RH) or from one of the [Cu–XH]^n+^ intermediates in Figure 4 or 5. Experimental determination of the hydrogen BDEs is rather complicated: it involves a thermodynamical cycle with measurement of pK_a_ and standard reduction potentials.[^114^, ^115^] With theoretical methods $\Delta E_{R-H}^{BDE}$ and $E_{{\left[Cu-XH\right]}^{n+}}^{BDE}$ can be calculated directly,^[72]^ which has been exploited to estimate the capacity for a given intermediate to participate in HAA reactions. A potential intermediate should have BDEs comparable to the relevant C−H bond of the substrate. This was estimated in Ref. [63] to be 422–433 kJ/mol (or 386–398 kJ/mol in terms of ΔG ). We comment on the BDEs in the discussion of the individual intermediates below. An interesting finding from calculated BDEs was that they only changed slightly with and without tyrosine present for several intermediates in Figures 4 and 5.^[63]^ The lack of the tyrosine in some LPMOs indicates that results between LPMOs with and without the tyrosine are transferable (cf. Figure 2). Moreover, deprotonation of tyrosine (investigated for **3 a**, **6 a** and **6 b**) also does not have a significant effect on the BDE.^[63]^

## 4 Reactive Intermediates

### 4.1 Superoxide and peroxide structures

The [CuO_2_]^+^ (**3 a**) intermediate can be described as Cu(II) bound to an O_2_⋅^−^ superoxide. The lowest spin‐state is a triplet with an open‐shell singlet (antiferromagnetic coupled Cu(II) and O_2_⋅^−^) energetically close.[^62^, ^64^, ^66^, ^67^, ^71^, ^72^, ^73^] The corresponding [CuO_2_] intermediate (**3 b**) can formally be described as a peroxide bound to Cu(II), but we refrain from speculating on the oxidation state in Figure 4.

The [CuO_2_]^+^ (**3 a**) intermediate was suggested as a candidate for the HAA reaction from the substrate in early (mainly experimental) studies.[^45^, ^91^, ^109^] However, so far all theoretical studies agree, that a CuO_2_‐species in the form of **3 a** or **3 b** is not sufficiently reactive. We have given a summary of the calculated reaction energies and barriers from both QM‐cluster and QM/MM studies in Table 2 (see further Table 1 for details of the setup in the individual studies). In addition to the numbers in Table 2, a QM‐cluster study by Hedegård and Ryde^[63]^ showed that the hydrogen BDE was 301.3 kJ/mol for the [CuO_2_]^+^ (**3 a**) intermediate. This is much too low for efficient HAA from the substrate. In this investigation, attempts to form the intermediate with a [CuO_2_] moiety (**3 b**) gave rise to a distorted structure and was not considered.^[63]^

**Table 2:** DFT reaction energies ($\Delta E_{react.}$ ) and barriers ($\Delta E_{TS}$ ) energies for the HAA from the substrate by [CuO_2_]^+^ (**3 a**) or [CuO_2_] (**3 b**) intermediates, given in kJ/mol.

Species	C	Functional	$\Delta E_{TS}$	$\Delta E_{react.}$
**3 a** ^[62]^	C${}_{1}$	B3LYP	146	117
**3 a** ^[62]^	C_1_	M06‐L	149	133
**3 a** ^[62]^	C_4_	B3LYP	156	131
**3 a** ^[64]^	C_1_	BP86	–	105
**3 b** ^[64]^	C_1_	BP86	–	129
**3 a** ^[66]^	C_4_	TPSS	156	140
**3 a** ^[66]^	C_4_	B3LYP	162	132
**3 a** ^[67]^	C_1_	TPSSh	146	–

Although the two intermediates, [CuO_2_]^+^ (**3 a**) and [CuO_2_] (**3 b**), are unlikely to be responsible for substrate oxidation, they may be important in the mechanism for hydrogen peroxide production,[^71^, ^73^, ^76^] which is a known side‐reaction for LPMOs in absence of substrate.^[124]^ The O_2_‐bound intermediates are further the only intermediates after reduction of Cu(I) in Figures 4 and 5 to be characterized by X‐ray or neutron diffraction.[^122^, ^123^] Although we are primarily interested in the HAA reaction, we briefly compare some of the reported optimized structures with [CuO_2_]^+^ (**3 a**) moieties as well as the experimental structures.

**Figure 6:**
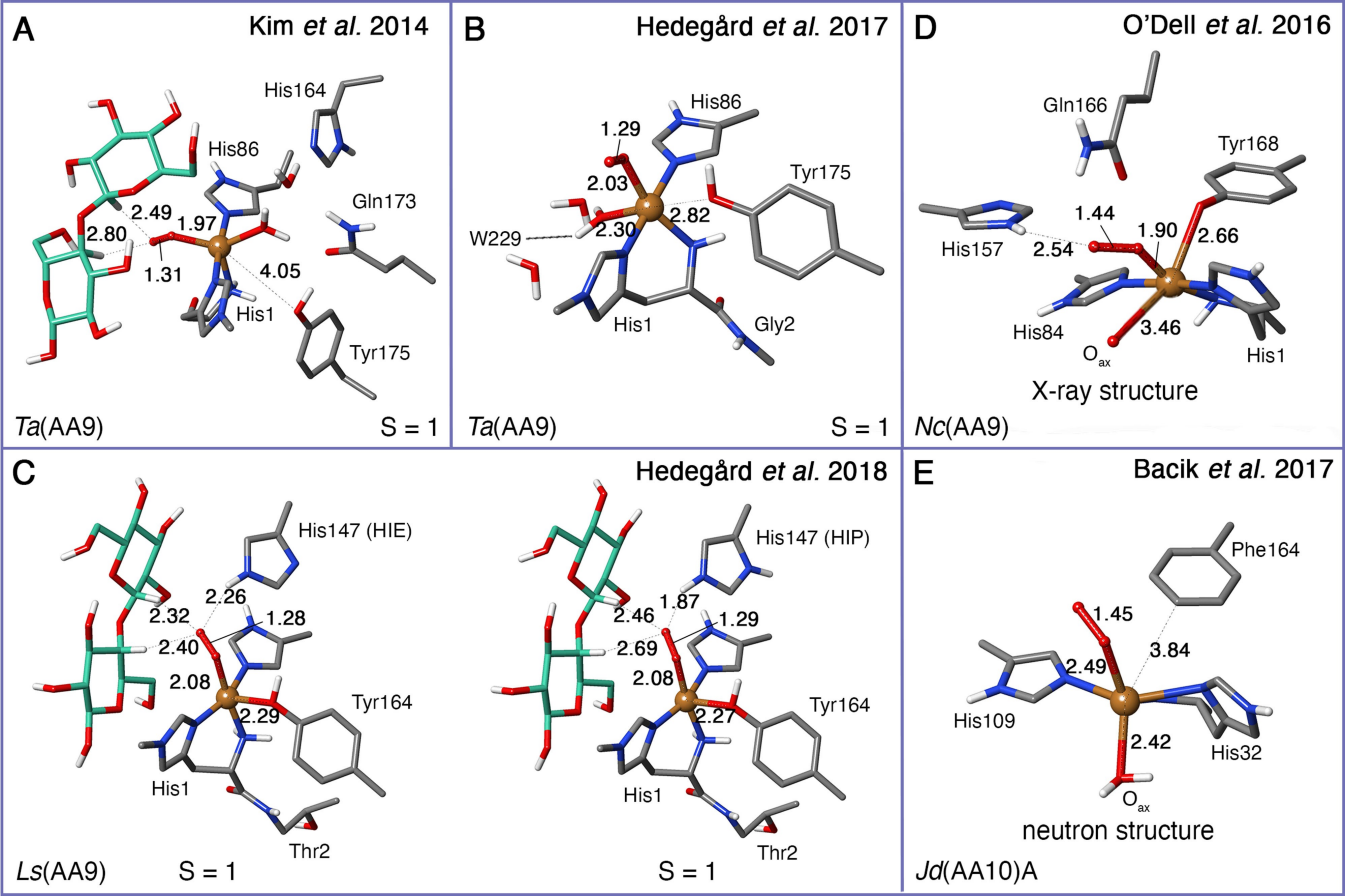
(A)–(C) QM regions from several theoretical investigations on the [CuO_2_]^+^ superoxide (**3 a**) for different LPMOs, where (C) also contains a substrate. The LPMO and the employed spin state are given at the bottom. For clarity, the hydrogen atoms are not displayed except for heteroatoms and C_1_/C_4_. (D)–(E): Crystallographic structures of the active sites of an AA9 LPMO (X‐ray, PDB: 5TKH chain A^[122]^) and an AA10 LPMO (neutron, PDB : 5VG1 chain B^[123]^), both showing a bound dioxygen species, modeled as peroxide (**3 b**). Selected distances are given in Å.

Structures of **3 a** have been optimized with both QM‐cluster and QM/MM methods, with examples of both shown in Figures 6A–C. We show the two published experimental structures[^122^, ^123^] in Figures 6D and E. Note that Figures 6A and C show the [CuO_2_]^+^ (**3 a**) optimized structures as part of an LPMO‐substrate complex, while the **3 a** intermediate in Figure 6B, ^[72]^ was optimized without substrate (the experimental structures in Figure 6D and E are also obtained without substrate). The computational, as well as the experimental structures, all agree on an end‐on bound dioxygen. The distance from the copper to the bound dioxygen is *∼*2.0 Å in all structures. Likewise, the O−O distance in the dioxygen is almost the same in all the optimized structures (Figures 6A−C). The X‐ray/neutron structures have somewhat longer O−O bonds, and are also modeled as Cu‐peroxide moieties[^122^, ^123^] (i.e., similar to **3 b** in Figure 4). However, the authors cannot conclusively rule out that the true nature is a superoxide. In case of the X‐ray structure, reduction from **3 a** may also occur due to photo‐reduction in the X‐ray beam. The largest differences among the optimized structures are seen for the tyrosine residue: the Cu−O distance between Cu and tyrosine is particularly large (over 4 Å) for the QM‐cluster study on *Ta*(AA9) by Kim et al.^[62]^ in Figure 6A. In QM/MM optimized structures for *Ta*(AA9)^[72]^ (Figure 6B), Cu−O distances are shorter (2.8 Å), and thus closer to the 2.7 Å in the X‐ray structure.^[122]^ It was discussed in connection with the first QM/MM study on LPMOs^[63]^ (without substrate) that the axial bonds in Cu complexes are weak and extremely flexible, making the distances vary significantly between different computational setups (further discussion of this issue can be found in Ref. [100] and references therein).

Another important difference between the [CuO_2_]^+^ (**3 a**) intermediate in Figure 6A and the other structures in Figure 6 is that **3 a** in Figure 6A has O_2_ in the axial position, relative to the tyrosine; this coordination was suggested based on a dioxygen species observed by Li et al.^[45]^ In a subsequent investigation with QM/MM,^[72]^ the [CuO_2_]^+^ (**3 a**) intermediate (in Figure 6B) was optimized with equatorial coordination of O_2_ and found to be 68 kJ/mol more favorable than axial coordination (we again refer to Table 1 for further details regarding the computational setup). The equatorial binding is a consequence of O_2_ replacing the equatorial water molecule of the resting state (Q_eq1_ in Figure 2B). All later investigations prefer this isomer (see for example Refs. [71, 72]), and this is also the isomer obtained in the X‐ray structure^[122]^ shown in Figure 6D. More details on equatorial/axial dioxygen binding are summarized in the review by Vu et al.^[42]^

Interestingly, the binding energy of O_2_ has been reported rather differently in different investigations: Kim et al.^[62]^ report values for O_2_ binding, i.e., formation of **3 a** (without the substrate), as downhill by −85 kJ/mol. Several different side‐on configurations were attempted but optimizations always ended in an end‐on configuration. The additional binding of substrate is further downhill by −22 kJ/mol.^[62]^ Another QM‐cluster investigation on *Ta*(AA9) without substrate employing the equatorial isomer^[71]^ found this value to be −3 kJ/mol. One difference between Refs. [62, 71] is that Ref. [71] employs a crystal structure with higher resolution as starting point. Additionally, a larger basis set compared to Ref. [62] was employed (Table 1). Meanwhile, large QM‐cluster calculations on *Nc*(AA9) – also with the equatorial isomer – found the binding energy of O_2_ to be −40 kJ/mol in the presence of substrate and −27 kJ/mol in absence of substrate.^[64]^

In the QM/MM study by Hedegård and Ryde,^[66]^ the [CuO_2_]^+^ (**3 a**) intermediate was investigated including the substrate, and with different protonation states and tautomeric forms of the second‐sphere residues (His147 in Figure 6C). The protonation state clearly has a large effect on the hydrogen bond between the Cu‐bound O_2_ and His147 as the H^ϵ^−O_2_^−^ distance changes from 2.9 Å to 2.3 Å upon protonation of the histidine. This histidine was also suggested to be involved in stabilization of a Cu−O_2_ species in the crystallographic work by O'Dell et al.^[122]^ and they obtain a H^ϵ^−O_2_^−^ distance of 2.5 Å, cf. Figure 6D.

### 4.2 Hydroperoxyl or hydroperoxo structures

The protonated forms of the [CuO_2_]^+^ (**3 a**) and [CuO_2_] (**3 b**) intermediates are denoted [CuOOH]^2+^ (**4 a**) and [CuOOH]^+^ (**4 b**) in Figure 4. Additional one‐electron reduction of **4 b** yields [CuOOH] (**4 c**) in step *(iv)c* (Figure 4). Few of these have been investigated systematically in HAA reaction with the substrate, but the investigations so far indicate that these intermediates are not sufficiently reactive: calculated hydrogen BDEs^[63]^ from QM‐cluster calculations of [CuOOH]^2+^ (**4 a**) were far below the substrate's BDE (317–324 kJ/mol, depending on whether tyrosine is included). In the same investigation, also [CuOOH]^+^ (**4 b**) was investigated but gave rise to a distorted structure (as described for **3 b** in previous the subsection). These findings are supported by Bertini et al.^[64]^ In their study on the *Nc*(AA9) LPMO, the attempted HAA from **4 a** led instead to protonation of a second‐sphere histidine (His160). The same authors also investigated **4 b** in HAA for the substrate and found the overall reaction to be thermodynamically feasible. However, no transition state could be located, and linear transit calculations suggest the reaction barrier to be too high in energy (126 kJ/mol). Similar conclusions were found for **4 c**. Thus, the theoretical studies published until now generally agree that intermediates with intact O−O bonds are not sufficiently reactive to abstract hydrogen from polysaccharide C_1_ or C_4_ atoms.

**Figure 7:**
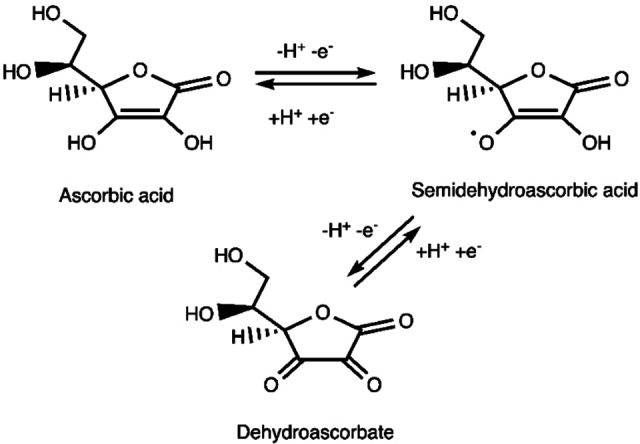
Oxidation of ascorbic acid in a series of proton transfer and electron transfer steps and/or proton‐coupled electron transfers.

It remains an open question whether the formation of the intermediates **4 a**–**4 c** is energetically feasible: from the experimental side, a complex with a [Cu−OOH] moiety was postulated to exist for inorganic model complexes, based on a comparison of measured and calculated UV‐vis spectra.^[125]^ In their QM‐cluster calculations, Bertini et al.^[64]^ compared formation of **4 b** from **3 a** to oxidation of ascorbic acid to semi‐dehydroascorbic acid (Figure 7) and found the total reaction to be slightly favorable.

In the QM/MM calculations reported by Hedegård and Ryde,^[66]^ attempts to optimize a [CuOOH] intermediate (**4 c**) by reduction of [CuOOH]^+^ (**4 b**) led to reduction of the second‐sphere histidine. Thus, the energetics of reaction *(iv)c* could not be obtained. However, reaction *(iv)a* in Figure 4, where the [CuOOH]^2+^ (**4 a**) intermediate is obtained from [CuO_2_]^+^ (**3 a**) could be calculated: the reaction was found to be feasible with a low barrier of 15–17 kJ/mol, and the reaction energy is between −6–0 kJ/mol, depending on the functional (B3LYP or TPSS).^[66]^ The reaction employed the second‐sphere histidine as proton donor (i.e., His147 for the investigated *Ls*(AA9) LPMO). The use of this residue as proton donor has been discussed intensively in recent literature: while the doubly protonated form may exist as a transient species, partially unrestrained refinement of high‐resolution AA9 X‐ray structures indicates that the second‐sphere histidine is in the HIE tautomer.^[126]^ The tautomeric form was confirmed by neutron diffraction in a recent article uploaded to *Chem‐Rxiv*.^[127]^ This article further suggests (based on MD simulations) that two conformers of this histidine exist in solution, but one of these is secluded in the crystal phase. This effectively excludes observation of a doubly‐protonated form from crystallography. The discussion of the histidine's role is also relevant for the further reactivity of the **4 a**–**4 c** intermediate in the O_2_ pathway of Figure 4 (see subsection below).

### 4.3 Oxyl and oxo intermediates

The intermediates in Figure 4 formed after the O−O cleavage (from either O_2_ or H_2_O_2_) are [CuO]^2+^ (**6 a**) and [CuO]^+^ (**6 b**), which can formally be described as Cu(II) bonded to oxo (O^2−^) or oxyl (O⋅^−^) ligands.[^62^, ^64^, ^65^, ^66^, ^67^] In the case of the [CuO]^2+^ (**6 a**) intermediate, one study^[64]^ has noted that **6 a** can be described as the resonance structure [Tyr−Cu(III)−O⋅^−^]↔[Tyr⋅^+^−Cu(II)−O⋅^−^], where analysis of the spin‐density shows that the latter resonance structure carries the main weight. Meanwhile, in the case of [CuO]^+^ (**6 b**), the d^9^ configuration on Cu(II) and the unpaired electron on the oxyl give rise to a triplet state, with an open‐shell singlet slightly higher in energy. For instance, the triplet ground state of [CuO]^+^ (**6 b**) intermediate obtained by Bissaro et al.^[67]^ has an open‐shell singlet state only 13 kJ/mol (TPSSh) or 15 kJ/mol (B3LYP) higher in energy. These values are quite similar to previously reported values in studies on AA9 LPMOs.[^62^, ^65^, ^66^] Due to the close‐lying spin‐states, both triplet and singlet potential energy surfaces should be investigated in theoretical calculations.

The [CuO]^2+^ (**6 a**) and [CuO]^+^ (**6 b**) intermediates can, as outlined previously, be formed from either O_2_ or H_2_O_2_ (cf. Figure 4). We discuss their formation and role in HAA reaction with the substrate in separate subsections below.

**Formation of oxyl and oxo intermediates**: The early QM‐cluster calculations by Kim et al.^[62]^ directly formed the [CuO]^+^ moiety (**6 b** without substrate) by adding 2H^+^ and 2e^−^ to the [CuO_2_]^+^ intermediate (**3 a**, also without substrate). The protons and electrons were added indirectly by employing a calculation on ascorbic acid (Figure 7) as reference, i.e., no barriers are calculated. They found the formation of **6 b** from **3 a** to be slightly uphill by 13 kJ/mol. The substrate is added after the formation of the [CuO]^+^ intermediate (yielding the **6 b** with substrate in Figure 4).^[62]^ Moreover, additional calculations led to two interesting conclusions: first, the entry point of the substrate does not alter the overall mechanism.^[62]^ Second, the N‐methylated histidine (His1 in Figure 6A) was found to be unimportant for the C−H abstraction. An experimental study^[40]^ on the *Ta*(AA9) LPMO concurs with these results as it showed that the methylation does not affect substrate specificity, redox potential, Cu binding, and the ability to activate O_2_. Thus, the role of the methylated histidine is still an open question.

The formation of the [CuO]^+^ (**6 b**) intermediate was also investigated with the QM‐cluster method by Bertini et al.^[64]^ They estimate the formation of **6 b** as a concerted form of reaction *(iv)c* and *(vi)d* in Figure 4, using oxidation of ascorbic acid to semi‐dehydroascorbic acid as reference (cf. Figure 7). With this reference, they estimate a reaction energy of −141 kJ/mol. They additionally investigated formation of **6 a** from reaction *(vi)a* in Figure 4. They obtain a stable structure; however, no reaction energies or barriers are given.

The QM/MM investigations[^65^, ^66^, ^67^] investigate barriers in all cases (as well as reaction energies) for the formation of the **6 b** intermediate. We collected the energetics in Table 3.

**Table 3:** DFT reaction energies ($\Delta E_{react.}$ ) and barriers ($\Delta E_{TS}$ ) for the formation of either **6 a** in reaction *(iv)a*, **5 b** in reaction *(v)b* and **6 b** in reaction *(v)c*, all referring to Figure 4. All energies are given in kJ/mol. His is the second‐sphere histidine (see Figure 2) – note that His is used as proton donor in reaction *(iv)a*. For further details to the employed computational setup, we refer to Table 1.

Species	Pathway	His	Functional	$\Delta E_{TS}$	$\Delta E_{react.}$
**6 a** ^[66]^	O_2_	HID	TPSS	48	−1
**6 a** ^[66]^	O_2_	HID	B3LYP	66	54
**5 b** ^[66]^	H_2_O_2_	HIE	TPSS	0^[a]^	−30
**6 b** ^[66]^	H_2_O_2_	HIE	TPSS	0^[a]^	−70
**6 b** ^[66]^	H_2_O_2_	HIE	B3LYP	0^[a]^	−74
**5 b** ^[65]^	H_2_O_2_	HIE	B3LYP	24^[b]^	−6^[b]^
**6 b** ^[65]^	H_2_O_2_	HIE	B3LYP	26^[c]^	−49^[c]^
**5 b** ^[67]^	H_2_O_2_	–	TPSSh	35	21
**5 b** ^[67]^	H_2_O_2_	–	B3LYP	54	31
**6 b** ^[67]^	H_2_O_2_	–	TPSSh	21	−51
**6 b** ^[67]^	H_2_O_2_	–	B3LYP	27	−52

[a] Reaction was found to proceed without barrier. [b] QM‐cluster values are 32 and approx. −20 kJ/mol (the latter is not given but based on Figure S3). [c] QM‐cluster values are 33 and 6 kJ/mol (open‐shell singlet).

In the QM/MM study by Hedegård and Ryde^[66]^ both O_2_ and H_2_O_2_ pathways are investigated; in the former, the [CuO]^2+^ (**6 a**) intermediate is formed in step *(vi)a* in Figure 4. The [CuO]^+^ (**6 b**) intermediate is formed by subsequent one‐electron reduction of **6 a** (step *(vi)b*), but no energies are given for this step. The proton required in step *(vi)a* is introduced at the second‐sphere histidine (His147), thus employing a HIP form of this histidine. With the proton from the second‐sphere histidine, the formation of **6 a** is feasible. However, the pathway is disfavored in comparison with formation of **6 b** from H_2_O_2_ through steps *(v)a*–*(v)c* in Figure 4 (see first four entries of Table 3). Note also that there is a significant functional dependence, where B3LYP yields a rather uphill reaction energy in the formation of **6 a** (H_2_O_2_ is favored, regardless of the functional employed). The O*−*O bond dissociation was also attempted from **4 a**, but this was rather unfavorable: the reaction was uphill 76 kJ/mol and had a barrier of 111 kJ/mol with the TPSS functional (this pathway is left out of Figure 4). We also mention that we have recently performed calculations on the same LPMO, but with a considerably larger QM region.^[128]^ These (yet unpublished) calculations show that both formation of the [CuO]^2+^ (**6 a**) intermediate through reaction *(vi)a* as well as formation of [CuO]^+^ (**6 b**) through reactions *(vi)c* and *(vi)d* are rather unlikely. A similar conclusion was obtained by QM/MM MD calculations on the *Ls*(AA9) H_2_O_2_ formation, occurring in absence of substrate.^[76]^ Here it was found that formation of **6 a** from **4 b** (with His147 as proton donor) is rather uphill and have a high barrier. The investigations in Refs. [66,76] employ different QM regions (see Table 1) making direct comparison difficult, but both investigations in fact agree for B3LYP, concerning the uphill reaction. Clearly, systematic investigation of the influence of both functionals and QM region sizes are highly requested.

**Figure 8:**
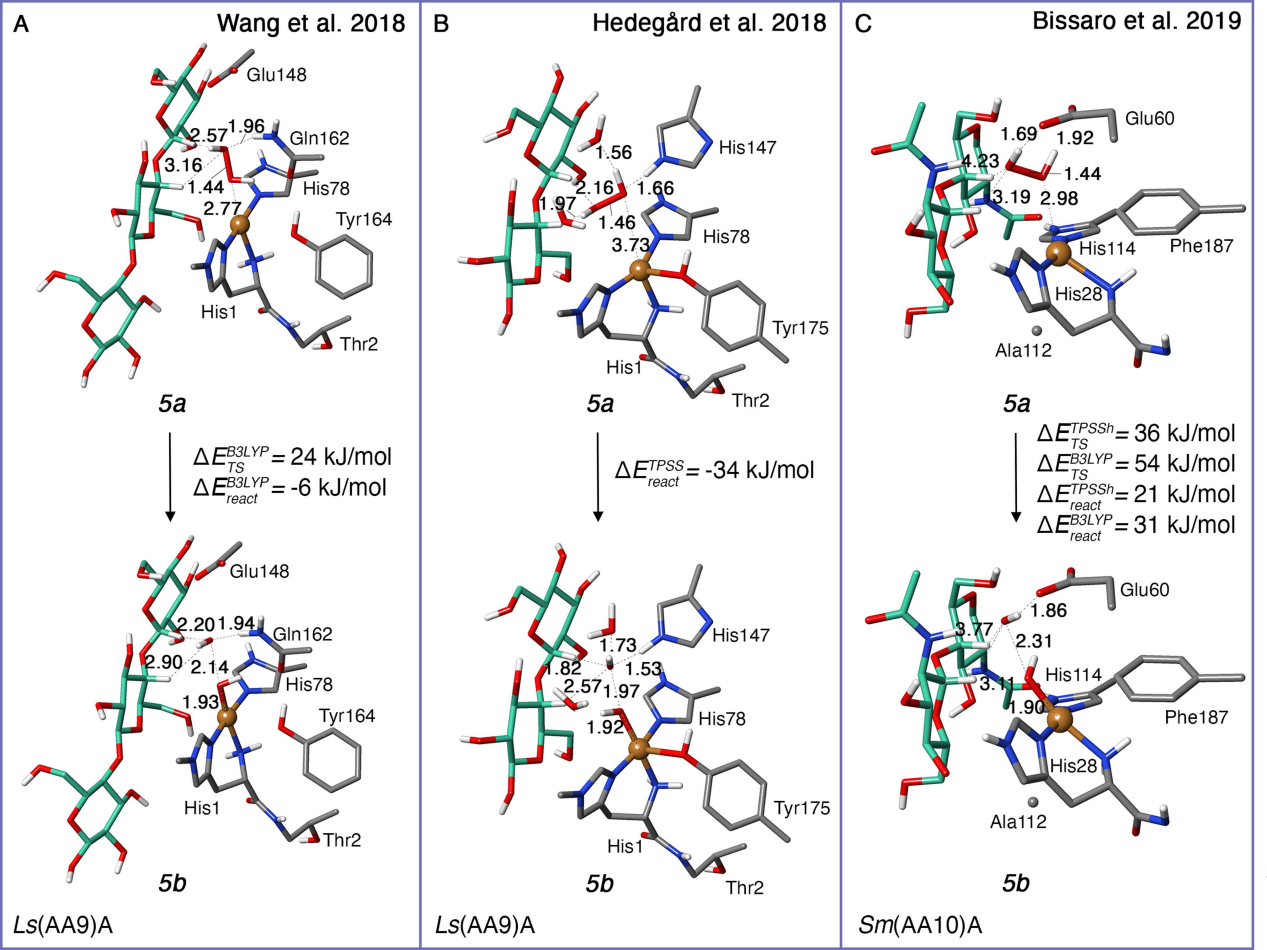
QM regions from several studies for the intermediates **5 a** and **5 b** from the H_2_O_2_‐driven pathway for different LPMOs. All structures are for the singlet state (S=0). For clarity, the hydrogen atoms are not displayed except for heteroatoms and C_1_/C_4_. Selected distances are given in Å. (B): Note that for (B) no transition state (hence not barrier) was found (see Ref. [66]). While energies in Ref. [66] generally are obtained with triplet *ζ* basis, no the energy for reaction (B) was provided. We provide the energy here, albeit only for a double *ζ* basis set. The reaction energies in (A) and (C) are obtained with a triplet *ζ* basis.

The QM/MM calculations by Wang et al.^[65]^ only investigate the H_2_O_2_ pathway, and also find this pathway to have low barriers (cf. Table 3, entries 5–6). It is interesting to compare these calculations to the QM/MM investigations by Hedegård and Ryde,^[66]^ since they employ the same underlying *Ls*(AA9) LPMO. However, the two investigations employ different DFT functionals and QM regions: a second‐sphere histidine (His147) is not included in Ref.,^[65]^ whereas a glutamine (Gln162) was not included in Ref. [66] We show the QM regions employed in the two studies in Figures 8A and B (see further Table 1).

Both investigations optimize a pre‐bound state corresponding to the **5 a** intermediate in Figure 4 in a singlet spin‐state. In Ref. [65], it is concluded (from both QM‐cluster and QM/MM calculations) that H_2_O_2_ is kept in the pocket between the substrate and the active site by interactions with the second‐sphere histidine and glutamine (Hie147 and Gln162 in Figures 8A and B). Thus, the peroxide does not coordinate Cu(I). A similar conclusion is reached in Ref. [66], although the two investigations obtain somewhat different orientations of H_2_O_2_ and distances to Cu (2.8 Å^[65]^ vs. 3.7 Å^[66]^ in Figures 8A and B). Both QM/MM investigations[^65^, ^66^] obtain the **5 b** intermediate as an OH⋅ radical with a long O−O bond to the hydroxyl in Cu(II)−OH. This was by Wang et al.^[65]^ denoted a caged OH‐radical. Both investigations find that **5 b** is an open‐shell singlet with a triplet lying only 13^[66]^ or 4^[65]^ kJ/mol above, respectively.

A larger difference is seen for the reaction energetics of reaction *(v)b* in Figure 4 (see Table 3 as well as Figures 8A and B). The study by Hedegård and Ryde^[66]^ obtained a reaction without a barrier, i.e., **5 b** formed spontaneously when H_2_O_2_ was moved towards the copper center. Meanwhile, Ref. [65] do find a TS for reaction *(v)b*, yielding a QM/MM activation energy of 24 kJ/mol and the reaction energy is Table 7 −6 kJ/mol. Notably, Wang et al.^[65]^ also investigated a different snapshot, yielding a similar value of 20 kJ/mol and −15 kJ/mol for activation and reaction energy, respectively.

The reaction in which the [CuO]^+^ (**6 b**) intermediate is formed from **5 b** (reaction *(v)c* in Figure 4) is also slightly different between Refs. [65, 66]: Hedegård and Ryde^[66]^ find that the [CuO]^+^ (**6 b**) intermediate spontaneously formed from **5 b** when H_2_O_2_ was attempted to be coordinated the Cu(I) ion in the triplet state; the reaction is considerably downhill as seen in Table 3. The **6 b** intermediate is most stable in the triplet state, i.e., reaction *(v)c* proceeds with a change in spin state. Meanwhile, Wang et al.^[65]^ obtain a low barrier and also a quite negative reaction energy for this reaction (cf. Table 3), employing the open‐shell singlet state. They also investigate the triplet state separately, finding similar values, although their triplet states are generally slightly higher in energy than their singlet states. Wang et al.^[65]^ further show that the caged OH radical (**5 b**) preferred to abstract a hydrogen atom from the Cu−OH moiety to form the [CuO]^+^ (**6 b**) species, rather than directly abstracting a hydrogen from the substrate (see Table 4).

**Table 4:** DFT reaction energies ($\Delta E_{react.}$ ) and barriers ($\Delta E_{TS}$ ) for the HAA from the substrate by intermediates after O−O bond dissociation (reaction *(vii)* in Figure 4). All energies are given in kJ/mol. His is the second‐sphere histidine (see Figure 2). For further details to the employed computational setup, we refer to Table 1.

Species	C	His	Functional	$\Delta E_{TS}$	$\Delta E_{react.}$
**6 b** ^[62]^	C_1_	HID	B3LYP	64	−13
**6 b** ^[62]^	C_1_	HID	M06‐L	76	2
**6 a** ^[64]^	C_1_	HID	BP86	62	−84^[a]^
**6 b** ^[64]^	C_1_	HID	BP86	26	−47^[a]^
**6 b** ^[66]^	C_4_	HIE	TPSS	104	−22
**6 b** ^[66]^	C_4_	HID	TPSS	69	−17
**6 b** ^[66]^	C_4_	HIE	B3LYP	111	−27
**6 b** ^[66]^	C_4_	HID	B3LYP	73	−22
**7 a** ^[66]^	C_4_	HIE	TPSS	103	57
**7 a** ^[66]^	C_4_	HID	TPSS	94	49
**7 a** ^[66]^	C_4_	HIE	B3LYP	93	−14
**7 a** ^[66]^	C_4_	HID	B3LYP	97	−22
**5 b** ^[65]^	C_4_	HIE	B3LYP	46^[b]^	−90^[b]^
**6 b** ^[65]^	C_4_	HIE	B3LYP	23	−40
**6 b** ^[67]^	C_1_	–	TPSSh	44	−40
**6 b** ^[67]^	C_1_	–	B3LYP	54	−47
**7 a** ${}^{'}$ ^[77]^	C_4_	HIE	TPSS	99	60
**7 a** ${}^{'}$ ^[77]^	C_4_	HIE	B3LYP	108	50

[a] Estimate as the product of HAA is not stable (see Ref. [64] for details). [b] The hydrogen in the glycosidic bond with the shortest distance to the OH‐radical was employed (see Figure 8A), i.e., C_1_−H was employed instead of C_4_−H.

Formation of the [CuO]^+^ (**6 b**) intermediate from hydrogen peroxide was also investigated with QM/MM for an AA10 LPMO in the study by Bissaro et al.,^[67]^ yielding essentially the same conclusion as Refs. [65, 66]. They start from a previous investigation^[74]^ of reaction *(v)a* in Figure 4, where classical MDs are used to show how H_2_O_2_ can enter the cavity between the active site and the bound substrate from the bulk solvent, forming **5 a**. In Ref. [67] they further employ umbrella sampling and find a free energy barrier of 8 kJ/mol for this reaction (from classical MDs). They optimize a structure similar to **5 a** with QM/MM (see Figure 8C), where H_2_O_2_ is hydrogen‐bound to the second‐sphere residues Glu60 and Asp185. The hydrogen bonding of particularly Glu60 is important and forces H_2_O_2_ into a distorted dihedral bond‐angle (53°, versus 113° in the gas‐phase equilibrium structure). The distance to Cu is rather different than for the studies on *Ls*(AA9) in Figures 8A and B, but in all cases, it is clear that H_2_O_2_ does not coordinate to Cu(I).

By employing MD with QM/MM on **5 a**, Bissaro et al.^[67]^ find that the O−O bond in H_2_O_2_ elongated (this occurs before 1 ns), consistent with the caged OH⋅ radical (**5 b**) obtained for *Ls*(AA9) in the QM/MM studies by Wang et al.^[65]^ and Hedegård and Ryde.^[66]^ The QM/MM optimized structure of **5 b** from Ref. [67] is displayed in Figure 8C. The elongated O−O bond is somewhat longer in the study on AA10^[67]^ (2.3 Å), compared to the two studies on *Ls*(AA9)[^65^, ^66^] (2.0–2.1 Å) in Figures 8A and B. The paper by Bissaro et al.^[67]^ is not completely clear on the open‐ or closed‐shell nature of this intermediate. Based on the high anti‐ferromagnetic coupling constant (given in their Supporting Information) we assume their **5 b** intermediate can essentially be interpreted as a closed‐shell singlet. The reaction energetics for reaction *(v)b* obtained by Bissaro et al.^[67]^ are provided in Table 3 and along with the structures of **5 a** and **5 b** in Figure 8C; they are obtained from QM/MM optimizations partly based on MD structures (cf. Table 1). By comparing the energetics in Figures 8A and B, we see that their barrier is slightly higher than found in Ref. [65] and significantly higher than the barrier in Ref. [66] Moreover, the reaction energy is somewhat uphill, compared to the downhill reaction energies in Refs. [65, 66].

Bissaro et al.^[67]^ further investigate reaction *(v)c* in Figure 4, forming intermediate [CuO]^+^ (**6 b**) from the **5 b** intermediate. The energies are also provided in Table 3, and the reactions are all feasible as in Refs. [65, 66] Similar to Ref. [66] (but unlike Ref. [65]), the reaction proceeds with a change from a singlet to a triplet potential energy surface.

In addition to the theoretical results, Bissaro et al.^[67]^ also report mutations of Glu60 according to Glu*→*Gln, Asn, Asp, and Ser. All mutations severely affected product yields (negatively), although substrate binding was not affected. Previous studies have found that a Glu*→*Ala mutation completely blocks reactivity.[^101^, ^129^] This residue is interesting since it is not present in the *Ls*(AA9) or the *Ta*(AA9)/*Nc*(AA9) LPMOs from previous investigations, but it is conserved in all AA10 C_1_ oxidizers. In Ref. [67], they denoted the Glu60 residue as structurally conserved^[44]^ across LPMOs as Glu or Gln. Therefore, they propose a similar role of Glu60 as to Gln162 and His147 in *Ls*(AA9). They speculate that His147 is normally in the HIE form but change to HID when the substrate binds. While the proposal is interesting, it should be noted that the comparison between HIE and HID forms with QM/MM^[66]^ always turned out to have HIE as the most stable tautomer and this concurs with the combined neutron diffraction and QM‐cluster investigation on the *Nc*(AA9)^[122]^ LPMO (although Ref. [122] did not include a substrate).

In conclusion, several theoretical investigations have now investigated formation of [CuO]^2+^ (**6 a**) and [CuO]^+^ (**6 b**), employing both AA9[^62^, ^64^, ^65^, ^66^] and AA10^[67]^ LPMOs. Initial QM‐cluster calculations predicted from reaction energetics that formation of **6 b** from O_2_ is feasible using the oxidation of ascorbic acid as a reference. However, these calculations did not include reaction barriers. Meanwhile, QM/MM calculations on *Ls*(AA9), show that formation of [CuO]^+^ (**6 b**) through one‐electron reduction of [CuO]^2+^ (**6 a**) (which is formed from a O_2_ ‐driven pathway) is disfavored compared to formation of **6 b** through **5 b** (which is formed from a H_2_O_2_‐driven pathway).^[66]^

Other QM/MM calculations employing either *Ls*(AA9)^[65]^ or an AA10 LPMO^[67]^ also find that the H_2_O_2_ pathway is feasible. However, the QM/MM investigations[^65^, ^66^, ^67^] differ on the details: all involve a pre‐bound state (**5 a**) in which second‐sphere residues are crucial in positioning H_2_O_2_ through hydrogen bonds. The **5 a** intermediate is converted to a caged OH radical (**5 b**), although the energetics are somewhat different. Surprisingly, the energies even differ for QM/MM investigations performed on the same underlying *Ls*(AA9) LPMO.[^65^, ^66^] The structures of intermediate **5 a** are also remarkably different. These differences may be caused by differences in the setup, such as the use of different QM regions.

Despite the different structures and energetics, the QM/MM studies all agree[^65^, ^66^, ^67^] that **5 b** is converted to **6 b** with a low[^65^, ^67^] (or vanishing^[66]^) barrier. Thus, it can be concluded that the theoretical investigations are converging to the conclusion that the O_2_ pathway is unfeasible. In comparison to experimental results, a recent kinetic investigation of *Ls*(AA9) concluded that this LPMO ”is unable to complete the catalytic cycle and cleave cellulose without H_2_O_2_”.^[130]^ Moreover, the involvement of the second‐sphere residues in positioning H_2_O_2_ for both AA9 and AA10 LPMOs can explain the importance of these residues known from site‐directed mutagenesis.[^43^, ^101^, ^129^]

**Figure 9:**
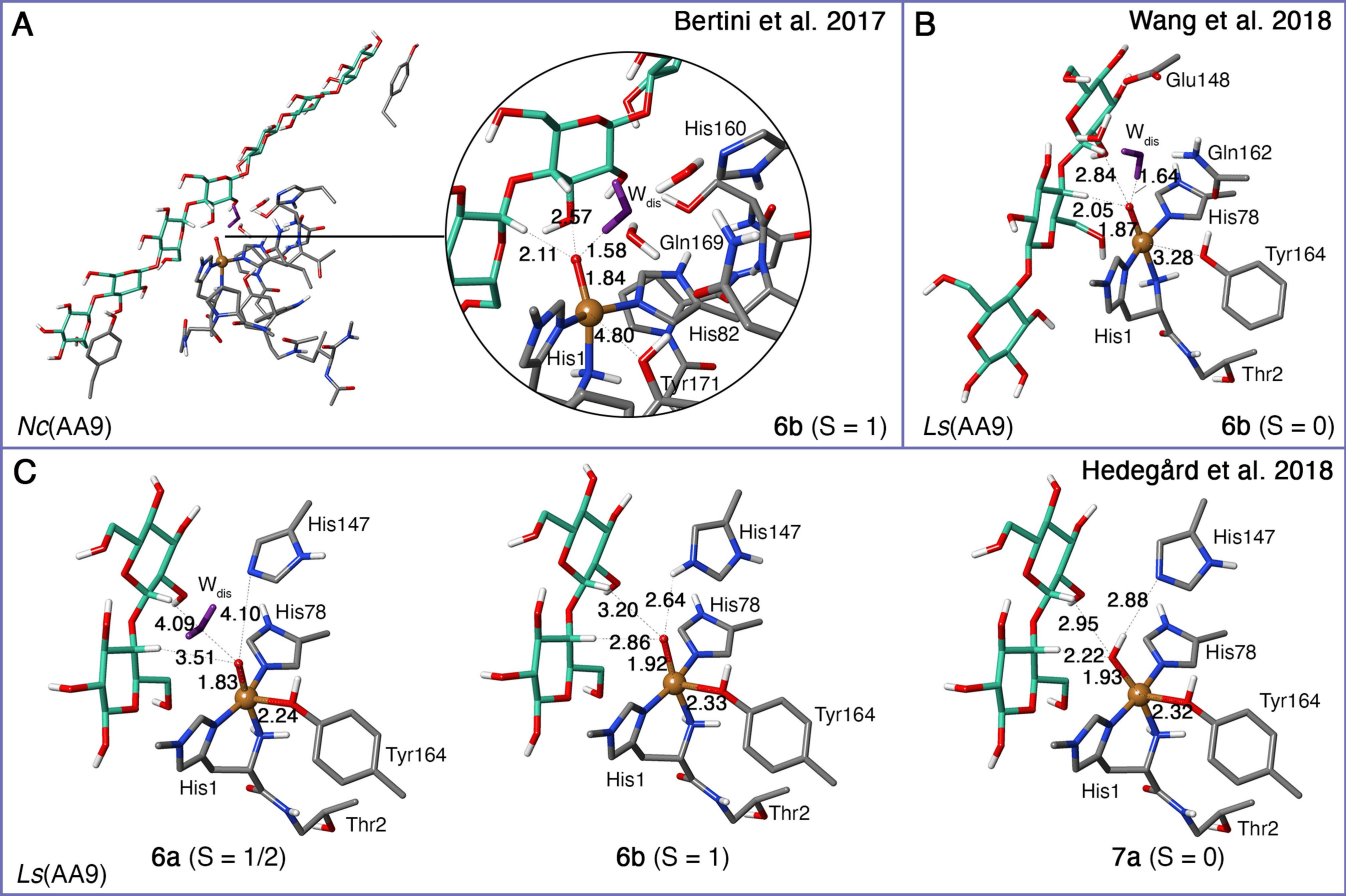
QM regions from several studies for oxyl **6 b** and related structures (**6 a**/**7 a**). For clarity, the hydrogen atoms are not displayed except for hetero atoms and C_1_/C_4_. Selected bond distances are given in Å. The water molecule formed after O−O cleavage (W_dis_, marked purple). Note that this water molecule is present in (C) for **6 a**, where it is formed in the O_2_ pathway, reaction *(vi)a* in Figure 4. It was removed in **6 b** from (C).

**Hydrogen atom abstraction and recombination reactions**: Calculated reaction barriers and energies for the HAA reaction with a polysaccharide substrate are compiled from various QM‐cluster and QM/MM investigations in Table 4. We additionally show selected, optimized structures for intermediates **6 a** and **6 b** in Figure 9. The values in Table 4 are clearly more favorable than for the intermediates with intact O−O bonds (cf. Table 2). The same conclusions were obtained from the calculation of BDEs:^[72]^ intermediates with [CuO]^2+^ (**6 a**) or [CuO]^+^ (**6 b**) moieties generally were found to have BDEs close to or higher than the BDEs for the substrate.

The QM‐cluster calculations are included in entries 1–4 of Table 4 (the remaining values are from QM/MM investigations). In these QM‐cluster calculations, Kim et al.^[62]^ and Bertini et al.^[64]^ both obtained favorable activation and reaction energies for the [CuO]^+^ (**6 b**) intermediate. However, the latter investigation obtained a considerably lower activation energy, presumably due to the different functional, cluster size, and underlying LPMO employed (see Table 1). Another difference between the two investigations is that the **6 b** intermediate optimized in Ref. [62] was an isomer with the oxyl (O⋅^−^) in a *trans* position, similar to the [CuO_2_]^+^ (**3 a**) intermediate from the same study (see Figure 6A). Meanwhile, Bertini et al.^[64]^ (as well as all other studies in Table 4) use the equatorial isomer. Bertini et al.^[64]^ further investigate HAA from the substrate for the [CuO]^2+^ (**6 a**) intermediate; they find a higher activation energy than for the [CuO]^+^ (**6 b**) intermediate, but the reaction is still feasible (cf. Table 4).

Moving to the QM/MM studies in Table 4, Hedegård and Ryde^[66]^ investigated HAA from the substrate with both HIE and HID tautomeric forms of the second‐sphere histidine (His147 for the employed *Ls*(AA9) LPMO, cf. Figure 9C). The results for the [CuO]^+^ (**6 b**) intermediate with different functionals are given in entries 5–12 in Table 4. While the form of the second‐sphere histidine can significantly impact the HAA barrier and reaction energy, the employed DFT functional is less critical for this particular reaction step. The barrier for the HAA from the substrate by the [CuO]^+^ (**6 b**) intermediate is higher, compared to the results from QM‐cluster calculations in Ref. [64] The lowest barrier is 63 kJ/mol (TPSS value) with the second‐sphere histidine in the HID tautomeric form. Employing the same tautomer of the histidine, Bertini et al. obtain 26 kJ/mol.^[64]^ However, both underlying LPMOs, the employed DFT functional, and the size of the QM cluster/QM‐region were quite different as seen from Figures 9A and C (see also Table 1). On the contrary, the QM/MM investigation by Wang et al.^[65]^ (entries 13–14 in Table 4 and Figure 9B) was performed on the *Ls*(AA9) similar to Ref. [66], but employing a different QM region and DFT functional. Yet, their barriers and reaction energies for the HAA reaction are closer to the QM‐cluster study in Ref. [64].

Part of the reason that the QM‐cluster study in Ref. [64] obtains similar energetics as the QM/MM study in Ref. [65], may be rooted in the underlying structures: both the **6 b** optimized by Bertini et al.^[64]^ (Figure 9A) and the **6 b** optimized by Wang et al.^[65]^ (Figure 9B) have a water molecule hydrogen bonding to the oxygen atom of the [CuO]^+^ moiety. The source of this water molecule is likely the O−O bond breaking step, i.e., either reactions *(vi)a* and *(vi)d* for the O_2_‐pathway or *(v)c* for the H_2_O_2_ pathway in Figure 4. This water molecule is not kept during calculations of the HAA reaction in Ref. [66] (cf. Figure 9C). This water molecule is also not seen in the QM‐cluster calculations by Kim et al.^[62]^

While comparing the Figures 9A−C, it can also be noted that the two studies leading to low HAA barriers[^64^, ^65^] (Figures 9A and B) also have very short distances to the abstracted C−H hydrogen (2.1 Å, compared to 2.9 Å in Figure 4C). Large structural variations are also seen for the axial tyrosine (Cu−O is between 4.8 and 2.3 Å), while the Cu−O bonds to the oxyl atom are rather similar (1.8–1.9 Å). Due to the previously mentioned similar BDE values with and without tyrosine,^[72]^ the large variation in distance to this residue is likely less important for the HAA energetics.

Another difference between Refs.[^64^, ^65^, ^66^] are the employed spin states: the HAA reaction calculated with QM/MM by Wang et al.^[65]^ is done on the singlet surface, with separate calculations on the triplet surface. The calculated barrier and reaction energies between the spin states are not markedly different, but no spin‐crossing appears to be observed, contrary to the two other QM/MM studies.[^66^, ^67^] For their small QM‐cluster study, Wang et al.^[65]^ state that they obtain a barrier of 41 kJ/mol for the HAA from the substrate, employing a triplet [CuO]^+^ (**6 b**) intermediate, which is reasonably close to the 23 kJ/mol in Table 4. They further state that a similar energy is obtained for the singlet‐energy surface.

The QM/MM investigation on an AA10 LPMO with a chitin substrate^[67]^ reports HAA reaction barriers and energies that are in between the ones found in the two QM/MM studies on *Ls*(AA9), cf. Table 4. The reaction proceeds through spin‐state crossing from **6 b** (triplet) to **6 c** (which they describe as an open‐shell singlet). The HAA with **5 b** was also attempted but found unfeasible. Note also that Ref. [67] keeps the water molecule hydrogen‐bonded to the [CuO]^+^ (**6 b**) oxyl atom, as discussed above.

The HAA abstraction is usually followed by a recombination step to form the hydroxylated product (see Figure 4). The barriers and reaction energetics associated with this reaction are compiled in Table 5, showing that the barriers are generally comparable to or lower than the corresponding HAA step. The corresponding reaction energies are in all cases much more downhill than the HAA step.

**Table 5:** DFT reaction energies ($\Delta E_{react.}$ ) and barriers ($\Delta E_{TS}$ ) for recombination reaction (reaction (*viii*) in Figure 4). Energies are given in kJ/mol. His is the second‐sphere histidine (see Figure 2). For further details to the employed computational setup, we refer to Table 1.

Species	C	His	Functional	$\Delta E_{TS}$	$\Delta E_{react.}$
**6 c** ^[62]^	C_1_	HID	B3LYP	34	−187
**6 c** ^[62]^	C_1_	HID	M06‐L	13	−166
**6 a** ^[64]^	C_1_	HID	BP86	0	−153^[a]^
**6 c** ^[64]^	C_1_	HID	BP86	0	−178^[a]^
**6 c** ^[66]^	C_4_	HIE	TPSS	40	−182
**6 c** ^[66]^	C_4_	HID	TPSS	35	−194
**6 c** ^[66]^	C_4_	HIP	TPSS	62	−158
**6 c** ^[66]^	C_4_	HIE	B3LYP	53	−193
**6 c** ^[66]^	C_4_	HID	B3LYP	44	−204
**6 c** ^[66]^	C_4_	HIP	B3LYP	53	−166
**6 c** ^[65]^	C_4_	HIE	B3LYP	28	−203
**6 c** ^[67]^	C_1_	–	TPSSh	11	−207
**6 c** ^[67]^	C_1_	–	B3LYP	8	−194

[a] Estimate as the product of HAA is not stable (see Ref. [64] for details). This also leads to a barrierless reaction.

**Figure 10:**
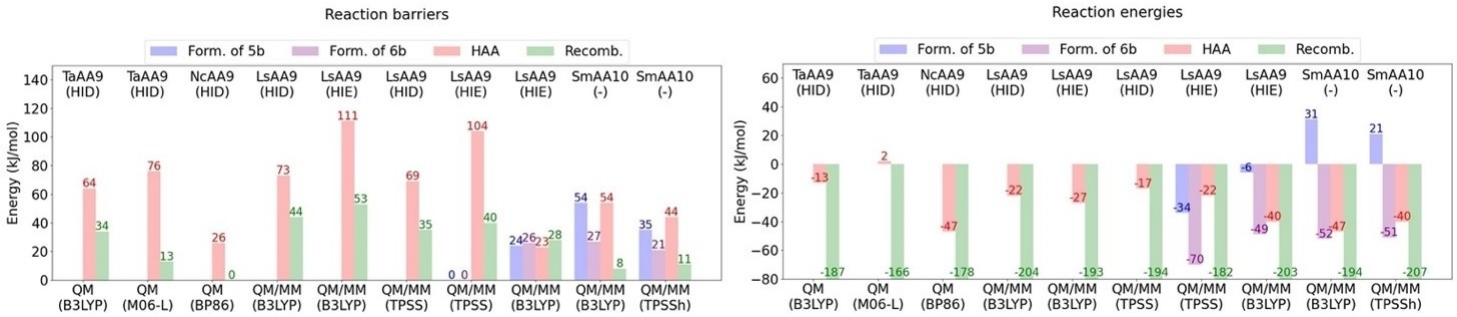
Comparison of reaction barriers and energies for the formation of **5 b**, formation of **6 b**, the HAA step, and the recombination step, see steps *(v)b*, *(v)c*, *(vii)* and *(viii)* in Figure 4. The values are from Tables 3 and 5 and include both different methods as well as different LPMO.

We conclude this section by comparing the calculated reaction barriers and energies of the HAA reaction in Table 3 (step *(vii)* in Figure 4) to the energies associated with formation of **6 b** through the peroxide pathway (steps *(v)b*–*(v)c*). The comparison is done in Figure 10, including both QM‐cluster and QM/MM investigations (and also the recombination step *(viii)* in Table 5, but this reaction is generally downhill and with a low barrier). The reaction energy for the HAA step is almost always downhill, but the large variations in the barriers (even among the QM/MM investigations) hinder general conclusions for a rate‐determining step.

Among the QM/MM investigations on *Ls*(AA9), either the energies of the steps are roughly equal,^[65]^ or the HAA step clearly has highest activation energy.^[66]^ For the investigation on the AA10 LPMO^[67]^ the order is generally step *(v)b* (formation of **5 b**)*≤*step *(viii)* (HAA)*<*step *(v)c* (formation of **6 b**)*<*step *(viii)* (recombination).

One potential reason for the differences is evident already in optimized structures, where distinct differences were apparent. Potentially, these differences are introduced by selecting different QM regions. At least, the distance between substrate C−H and the [CuO]^+^ (**6 b**) oxyl correlate with the HAA barrier, as discussed above. Systematic investigations on the QM region size will be required to quantify the origin of the differences. Moreover, most investigations rely on a few structures, and including dynamics to a greater extent will be essential in future investigations.

None of the intermediates in Tables 3, 4, 5 have been observed. One link to experiment is conversion of measured rate constants to a reaction barrier (*ΔE*_TS_) by using equation 2

(2) $${\;\;k}_{cat}=A\;exp\left(\frac{{\Delta E}_{TS}}{RT}\right)\;$$

where *R* is the gas constant, *T* is the temperature and *A* is denoted the pre‐exponential factor. Unfortunately, this conversion is not unproblematic: the kinetic parameters are difficult to measure experimentally for LPMOs (see Ref. [57] for a review). Moreover, the rate constant depends significantly on the reaction conditions of a given LPMO (a summary of kinetic data is given in the review by Chylenski et al.^[17]^). However, even with an accurate measurement, the experiment barrier is difficult to convert to a theoretical barrier since the enzyme often undergoes several side reactions. Moreover, the pre‐exponential factor is not known, and different values have been employed. Experimental rate constants are collected in Table 6 along with resulting barriers with different pre‐exponential factors (the factors are taken from recent QM/MM investigations[^65^, ^66^, ^67^]). From these values, it seems that calculated barriers below 30–40 kJ/mol are too low. This argument was used by Wang et al.^[65]^ to conclude that better correspondence between theory and experiment is obtained if hydrolysis of the substrate in water is considered rate determining. Unfortunately, the calculation of the hydrolysis step was done with a rather different computational setup, making it difficult to compare with the HAA reaction.

**Table 6:** Experimentally derived rate constants ($k_{cat}$ in s^−1^) for substrate oxidation and barriers ($\Delta E_{TS}$ in kJ/mol) for chosen pre‐exponential factors (A in s^−1^). All rate constants were obtained under different reaction conditions not explicitly stated here (see Ref. [17] for a summary), but O_2_ and H_2_O_2_ refer to the (apparent) co‐substrate that was employed.

		$\Delta E_{TS}$		
Condition	$k_{cat}$	A=$6\times 10^{12}$ ^[a]^	$10^{12}$ ^[b]^	$0.23\times 10^{11}$ ^[c]^
H_2_O_2_	6.7^[131]^	68	64	54
O_2_	0.17^[47]^	77	73	64
O_2_	0.28^[47]^	76	72	62
O_2_	0.11^[27]^	82	77	67

[a] Factor used in Ref. [66] to compare results to kinetic data. [b] Factor used in Ref. [65] to compare results to kinetic data. [c] Factor obtained in Ref. [67] by fitting results the calculated rate determining step (HAA from the substrate) to the $k_{cat}$ of 6.7 s^−1[131]^ of the H_2_O_2_‐driven catalysis by *Sm*(AA10).

### 4.4 Hydroxyl or hydroxide intermediates

In this final section, we discuss hydroxyl or hydroxide intermediates with a [CuOH]^2+^ moiety, whose formation is shown as **7 a** or **7 a′** in Figure 5.

The QM‐cluster calculations estimate the hydrogen BDEs^[63]^ for the [CuOH]^2+^ (**7 a**) complex as well as for the more exotic [CuOH]^3+^ (not shown in Figure 5), to be sufficiently potent for HAA from a substrate. However, the calculated BDEs displayed a large functional‐dependence, making it difficult to give a firm conclusion. The calculated BDEs for [CuOH]^2+^ (**7 a**) were 387 kJ/mol with the TPSS functional, whereas the corresponding BDE with B3LYP is 458 kJ/mol. The BDEs for [CuOH]^3+^ were higher at 404 kJ/mol (TPSS) and 463 kJ/mol (B3LYP).

The [CuOH]^2+^ (**7 a**) intermediate was also investigated by QM/MM in the study by Hedegård and Ryde,^[66]^ where barriers and reaction energies for HAA with the substrate were calculated (cf. Table 4): On the one hand, the barrier is not very functional dependent with values between 93–103 kJ/mol, depending on the functional. On the other hand, the reaction energy is much more dependent on the employed functional: the TPSS functional yields a reaction energy that is rather uphill (between 49–57 kJ/mol), whereas B3LYP yields a downhill reaction (between −14 and −22 kJ/mol). The reason for the functional dependence was in Ref. [66] ascribed to the change in spin‐state (from singlet to triplet) during the HAA reaction. Indeed, a later investigation^[68]^ found [CuOH]^2+^ (**7 a**) to be a closed‐shell singlet with DFT, but the triplet‐singlet splitting was very sensitive to the employed functional (the singlet state of the **7 a** intermediate was also found to be 53 kJ/mol more stable than the triplet with QM/MM in Ref. [66]). Perhaps more ominous is that none of the DFT results were close to the spin‐state splitting obtained with a highly accurate wave function.^[68]^

The formation of the [CuOH]^2+^ (**7 a**) intermediate was also investigated with QM/MM in Ref. [66] The mechanism outlined in Figure 5 is through protonation of [CuOH]^+^ (**6 b**) to **7 a**, i.e., employing the second‐sphere histidine as proton donor (this corresponds to His147 in Figure 9C). This was in Ref. [66] calculated to be a favorable process; the reaction energy is −148 kJ/mol (with both TPSS and B3LYP) and the activation barrier is low: 19 kJ/mol with TPSS and 40 kJ/mol with B3LYP. It can therefore be speculated whether **6 b** is in equilibrium with **7 a**. Moreover, Ref. [66] also investigated a scenario where the second‐sphere histidine (His147 in Figure 9) is doubly protonated. In this case, [CuOH]^2+^ (**7 a**) formed spontaneously from **5 b**, regardless of the spin state of **5 b**.

In a very recent study, McEvoy et al.^[77]^ investigated an alternative mechanism for protonation of [CuO]^+^ (**6 b**): as outlined in Figure 5, a [CuOH]^2+^ (**7 a** ${}^{'}$ ) moiety can also be formed from proton transfer from the coordinating tyrosine (although it is more likely an actual hydrogen atom transfer). McEvoy et al.^[77]^ calculated the barrier and reaction energies for HAA from the substrate with both the TPSS and B3LYP functionals (cf. Table 4). The barriers were in fact comparable to the **6 b** and **7 a** intermediates, but reaction energies were consistently rather uphill with both functionals. It was therefore concluded that **7 a** ${}^{'}$ was more likely an intermediate involved in a protective mechanism, obtained when **2** reacts with H_2_O_2_ without substrate present. This was concluded based on the fact that the formation of **7 a** ${}^{'}$ was found to be feasible from **6 b**, also without substrate: the barrier was 53 kJ/mol and a reaction energy of −70 kJ/mol with TPSS (the corresponding B3LYP numbers were 64 and −48 kJ/mol, respectively).

## 5 Conclusion and Outlook

Lytic polysaccharide monooxygenases (LPMOs) oxidize glycosidic bonds in polysaccharides, thereby boosting the degradation of cellulose and other recalcitrant polysaccharides. Their mechanism has been intensively investigated since their original discovery in 2010; here we have given a critical review, focusing on the theoretical investigations of the oxidation of the substrate along with a comparison with selected experimental investigations for key steps.

The LPMOs employ either O_2_, H_2_O_2_, or both as co‐substrate. The recent theoretical works have shown that the formation of an intermediate sufficiently reactive to oxidate the substrate is feasible with H_2_O_2_ as substrate. Thus, investigations now converge towards a H_2_O_2_‐driven mechanism. This mechanism alleviates the need for exogenous electrons and protons, whose delivery is difficult to explain with the substrate blocking the entry to the active site. As an increasing number of experimental investigations also support the use of H_2_O_2_ as co‐substrate, it begs the question: are LPMOs really monooxygenases?

For the actual rate determining step, most theoretical studies have investigated an oxyl intermediate with a [CuO]^+^ (**6 b**) moiety for the HAA step. A few investigations have noted that an intermediate with a [CuOH]^2+^ moiety may also be sufficiently reactive. Yet, calculated reaction energetics for the HAA step as well as structural parameters for involved intermediates differ vastly across theoretical investigations. This is the case even when the same underlying LPMO is employed. One issue may be that all investigations employ density functional theory (DFT), and some of the reaction steps are rather dependent on the employed functional. Another issue may step from the well‐known dependency of the QM‐region size in QM/MM calculations. Unfortunately, the employed QM regions have differed significantly, presumably leading to some of the observed differences. Future studies should focus on how to select a proper QM region and may also need to include dynamics to a larger extend. Moreover, investigations should also attempt to employ theoretical methods beyond DFT.

Another direction recently taken is to trap reaction intermediates and employ electronic spectroscopy and calculations to link their geometrical and electronic structures.[^77^, ^110^, ^111^, ^132^] The intermediates detected so far were, however, concluded to not be part of the substrate oxidation. Yet, the method may become important for detecting the oxidative intermediate in future explorations.

Finally, we note that theoretical investigations of the oxidative mechanism have exclusively been concerned with AA9 and AA10 LPMOs. It remains an open question how general the mechanisms in Figures 4 and 5 are across the LPMO families. Along these lines, to the best of our knowledge, theoretical work with LPMOs and hemicellulases such as xylan has not been carried out, even though hemicellulases comprise a large part of plant cell walls.^[133]^

## Conflict of interest

The authors declare no conflict of interest.

6

## Biographical Information

*Marlisa M. Hagemann was born in Hamburg (Germany). She started her research in Erik D. Hedegård's group in 2020 at Lund University. After receiving her M.Sc. degree in Chemistry from Kiel University, she joined the Hedegård group as a Ph.D. student at the University of Southern Denmark in 2022. She focuses on the study of metalloenzymes using computational methods*.

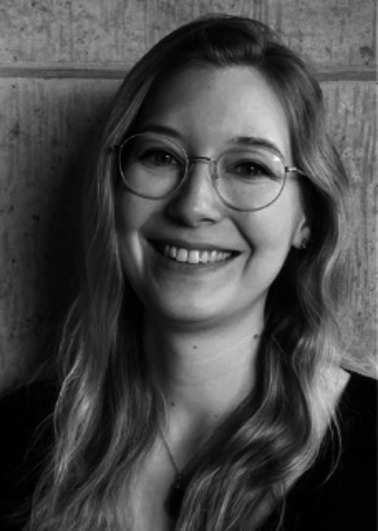

## Biographical Information

*Erik D. Hedegård was born in Copenhagen (Denmark). He got his Ph.D. degree in the group of Jacob Kongsted, University of Southern Denmark on polarizable embedding methods in 2014. From 2014–2016 he was a post‐doc at ETH Zürich (Switzerland) and then (2016–2018) at Lund University (Sweden). During these years, he worked with density matrix renormalization group methods, as well as QM/MM for metalloenzymes. He started a group in 2018 at Lund University and in 2020, he accepted the position as associate professor at the University of Southern Denmark*.

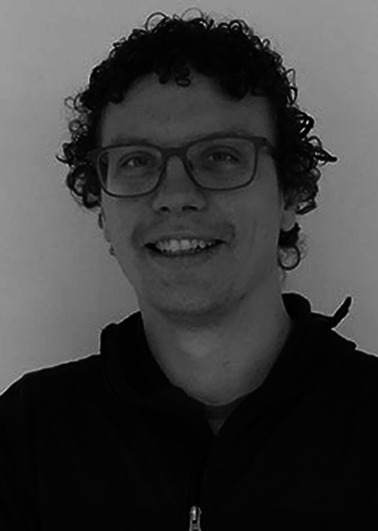

## Data Availability

The manuscript is a review without substantial amount of new data.
